# Replication-Associated Mutational Pressure (RMP) Governs Strand-Biased Compositional Asymmetry (SCA) and Gene Organization in Animal Mitochondrial Genomes

**DOI:** 10.2174/138920212799034811

**Published:** 2012-03

**Authors:** Qiang Lin, Peng Cui, Feng Ding, Songnian Hu, Jun Yu

**Affiliations:** CAS Key Laboratory of Genome Sciences and Information, Beijing Institute of Genomics, Chinese Academy of Sciences, 100029 Beijing, China

**Keywords:** Function-based selection, mitochondrion genome, replication-associated mutational pressure, strand-biased compositional asymmetry.

## Abstract

The nucleotide composition of the light (L-) and heavy (H-) strands of animal mitochondrial genomes is known to exhibit strand-biased compositional asymmetry (SCA). One of the possibilities is the existence of a replication-associated mutational pressure (RMP) that may introduce characteristic nucleotide changes among mitochondrial genomes of different animal lineages. Here, we discuss the influence of RMP on nucleotide and amino acid compositions as well as gene organization. Among animal mitochondrial genomes, RMP may represent the major force that compels the evolution of mitochondrial protein-coding genes, coupled with other process-based selective pressures, such as on components of translation machinery— tRNAs and their anticodons. Through comparative analyses of sequenced mitochondrial genomes among diverse animal lineages and literature reviews, we suggest a strong RMP effect, observed among invertebrate mitochondrial genes as compared to those of vertebrates, that is either a result of positive selection on the invertebrate or a relaxed selective pressure on the vertebrate mitochondrial genes.

## INTRODUCTION

The variation of genome compositions, nucleotide or amino acid sequences, records the evolutionary history of these genomes over evolutionary time scale [1, 2]. One of the most outstanding case is the variation of G+C content that occurs universally, found in all forms of genomes, including mammals, plants, and bacteria [3-7]. The early notion about unbalance genomic composition is discovered in bacteria, and genomic CG content variation has been thought to be closely related to environmental pressure and evolutionary state [8-10], and CG content asymmetry between the two DNA strands (positive *vs*. negative, leading *vs*. lagging, and Watson *vs*. Crick) is associated with either replication- or transcription-associated mutations [11, 12]. In return, dynamic nucleotide composition also shapes codon usage, transcription and translation efficiencies, and therefore affects strand-biased gene organization [13, 14]. By using such logic, methods have been developed to predict origin of replication [15, 16]. In vertebrates, especially in mammals and birds, a long-range mosaic GC content variation spanning hundreds of kilobases was termed “isochores” [17, 18]. Although it is rather controversial to explain the causative mechanism about isochores, such a structure may reflect a clustering nature for some GC-rich genes rather than a global selection or mutation signature. Among the transcripts of protein-coding genes, there is a transcription-associated DNA mutational spectrum that is transcript-centric and exhibits a negative GC content gradient from 5’ to 3’ along the orientation of transcripts, and such feature becomes more pronounced among worm-blooded vertebrate and grass genes [19, 20]. GC-skew is therefore useful in predicting transcript start sites among plants [21]. Nevertheless, strand-biased nucleotide composition changes of diverse genomes are complex as mutations are always at work and selections act at each level of functional networks, all the way down to individual genes and their regulatory elements. A relative straightforward system, other than prokaryotic genomes, is the animal mitochondrial genome that is highly conserved and small in size [22, 23]. 

Up to now, there have been over 1,500 mitochondrial genomes sequenced from different taxonomic groups of animals. Such a significant collection provides a significant resource for large-scale comparative analysis. Since metazoan mitochondrial genomes are not likely to recombine, their gene structures are rather stable and the number of genes remain constant, providing a model for the study of sequence evolution and compositional dynamics [24]. In this review, we focus our discussions on genome compositional dynamics of mitochondrial genomes to illustrate the evolutionary relationship between genomic DNA composition variation and its functional consequences.

## THE STRAND-BIASED NUCLEOTIDE COMPOSITIONS 

**Fig. (1):**
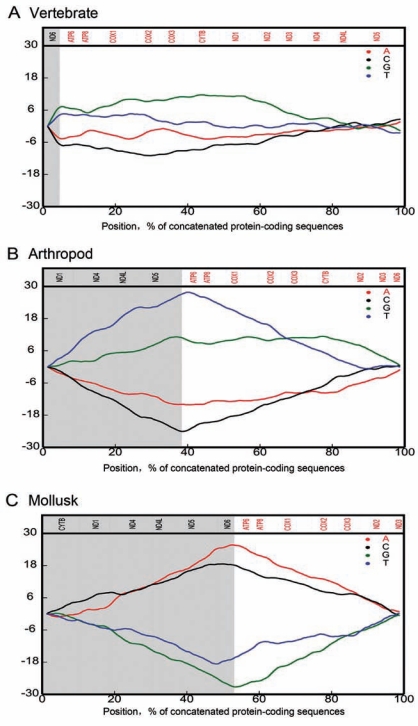
Nucleotide compositional analysis on concatenated 13 mitochondrial protein-coding sequences of different animal taxa: **A**, *Orycteropus afer*, a representative of vertebrates; **B**, *Bactrocera papaya*, a representative of arthropods; and **C**, *Katharina tunicata*, a representative of mollusks. Numbers on the y-axis indicate relative abundance of the four nucleotides. The H- or L-strand genes are divided with shading. The genes (top) high-lighted in red are those transcribed in sense direction from L-strand. The genes marked in black are those transcribed in sense direction from H-strand.

The two DNA strands of animal mitochondrial genomes have different buoyant densities, and are thus named as heavy strand (or H-strand) and light strand (or L-strand). The two strands often have different nucleotide compositions, where H-strand is GT-rich and L-strand is AC-rich. Such a difference has been explained based on the strand-displacement model of mitochondrial DNA (mtDNA) replication mechanism. During mtDNA replication, the parental H-strand, as a single DNA strand, has a longer single-stranded state, serving as template for the daughter L-strand synthesis [25-27]. Since spontaneous deamination of both A (adenine) and C (cytosine) [28-30] occurs frequently in single-stranded DNA [31], it essentially leads to strand-biased composition. The deamination of A leads to I (hypoxanthine), forming stronger base pairing with C than with T (thymidine) and generating an A:T→G:C mutation. The deamination of C leads to U, generating C:G→U:A mutation. Once the C→U mutant-bearing strand is used as a template to replicate the daughter L-strand, it leads to a G→A mutation in the L-strand after one round of DNA duplication. Therefore, the H-strand, left single-stranded for an extended period of time during DNA replication, tends to accumulate A→G and C→U mutations and become rich in G and T, and meanwhile, the H-strand accumulates an excess of G over C and T over A, i.e. GC skew and AT skew, whereas the L-strand becomes rich in A and C, showing an excess of C over G and A over T (Fig. **1**).

## THE ASYMMETRY OF PROTEIN-CODING SEQUENCES 

In protein-coding sequences, the effect of strand-biased compositional asymmetry differs at different codon positions [32]. Because of the wobble position (the 3rd codon position or cp3) and codon redundancy, the accumulative consequence of directional mutation pressure can be observed readily based on analysis of the nucleotide composition at cp3 sites of fourfold degenerate amino acids. We collected 870 vertebrate and 342 invertebrate complete mitochondrial genome sequences from different taxonomic groups archived at NCBI and calculated the relative synonymous codon usage (RSCU) as a measure of codon usage bias, which is defined as the observed frequency of a codon divided by the expected frequency under the assumption of equal codon usage (Table **1**). Among the vertebrate mtDNAs, the L-strand genes tend to end their codons with A or C more frequently than G or T as compared to the H-strand genes that prefer to have codons ending with G and T. It is obvious that genes of the two strands exhibit distinct codon usages unique to the strand. In contrast to vertebrates, arthropod mtDNAs share similar mutation bias in the sites of four-fold or six-fold degenerate amino acids but it weakens in the two-fold degenerate sites, or rather their corresponding amino acids, are very sensitive to transversions (purine to pyrimidine or *vice versa*) that always change the amino acid sequences. 

Among animals, mitochondrial genomes, with a few exceptions, encode 22 tRNA genes, resulting one tRNA species per amino acid on average (Table **1**). The exceptions are leucine and serine tRNAs, which are two of the three six-fold degenerate amino acids and among the most abundant amino acids of protein-coding sequences (the case of the other six-fold degenerate amino acids Arg is rather complex in mitochondrial genomes so we chose not to discuss it in details). There are usually two tRNAs for their six codons and only one (occasionally two) for each amino acid; the first two codon positions (codon positions 1 and 2, or cp1 and cp2) are complementary to its tRNA anticodons and the third one, cp3, involves wobble base pairing. Moreover, all anticodons of 22 tRNAs are highly conserved, which perfectly match the single codons ending in A or C (Fig. **S1**). The most frequently used codons by the L-strand genes are those perfectly match to their tRNAs, whereas for the H-strand genes, the most frequently used codons do not perfectly match to their tRNAs. There is only one gene found to be on H-strand among almost all vertebrate mitochondria is *nad6*. A recent study found that in Antarctic notothenioids, *nad6, *adjacent to tRNA-Glu, has translocated into the control region from their canonical location to release H-strand’s mutation pressure [33]. In addition, a study on horses residing at different altitudes provided evidence that their *nad6* genes have the lowest genetic diversity and may undergo purifying selection for adapting to high altitudes, and again the observation suggests that *nad6* may become intolerable to additional mutations [34]. Therefore, there may be an advantage for genes to have better matched codons and anticodons for a “best-fit” in the protein translational machinery, and an appropriate positioning on the right strand alters the strand distribution of mtDNA genes. 

## THE ASYMMETRY OF AMINO ACID COMPOSITIONS

In bacteria, asynchronous replication between leading and lagging strands induces SCA that shapes codon and amino acid usages and contributes to strand-biased gene distribution (SGD) [13, 14, 35, 36]. In *E. coli *and *Bacillus subtili*, Gly, Val, Glu, and Asp are relatively abundant on leading strand but Ile, Thr, and His are relatively more abundant on lagging strand. In addition, hydrophilic amino acids are more plentiful in proteins encoded by regions close to the terminus of chromosome replication, whereas hydrophobic amino acids are more abundant in proteins encoded by regions close to the origin of the replication [37]. Such replication-associated amino acid compositional asymmetry is also found among metazoan mitochondrial genomes. Compared with CG-skew and AT-skew in flatworms and mammals, an opposite SCA was observed in amino acids of protein-coding sequences [38]. 

**Fig. (2):**
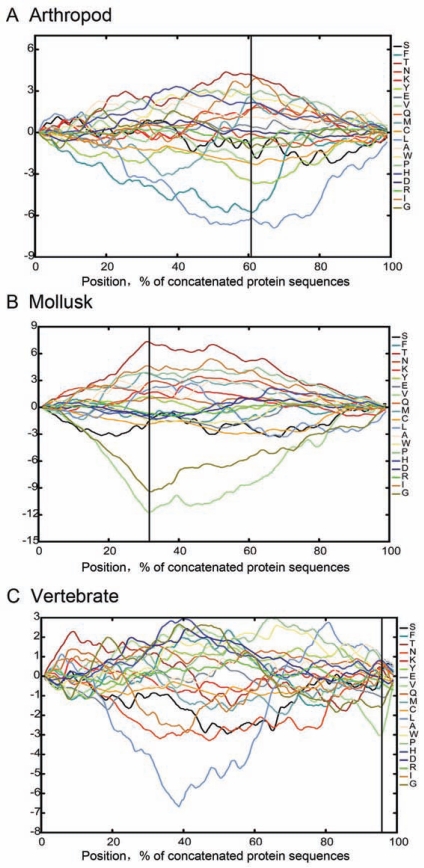
Amino acid compositions along concatenated 13 mitochondrial protein sequences from diverse animal lineages: **A**, *Pyrocoelia rufa*, a representative of arthropods; **B**, *Lampsilis ornate*, a representative of mollusks; and **C**, *Trichosurus vulpecula*, a representative of vertebrates. Numbers on the y-axis indicate relative amino acid abundance. The vertical lines divide the L- and H-strand genes.

To summarize the impact of RMP on amino acid compositional dynamics, we used dinucleotide frequency to show amino acid composition variations between the two strands, and *Pyrocoelia rufa*,* Lampsilis ornate, *and *Trichosurus vulpecula*, are representing arthropods, mollusks, and vertebrates, respectively (Figs. **2** and **S2**). In *P. rufa* and *L. ornate* mitochondrial genomes, AT, AA, AC, CC, and GC are used more frequently on their L-strands, as opposed to TT, TG, GT, and GG used more frequently on their H-strands. As a result, Thr, Ile, Pro, Ala, and Lys are more frequently found among the L-strand genes, whereas Gly, Val, Phe, and Cys are more prevalent among the H-strand genes. This amino acid composition bias is obvious among invertebrate mtDNAs. Despite the fact that vertebrate mtDNA harbor one gene, *nad6*, on their H-strand, and the pattern is not statistically significant, we can still see more frequent Val, Gly, and Glu on the H-strand of *T. vulpecula* mtRNA. In addition, the synonymous sites of four-fold and six-fold degenerate amino acids are more strongly affected as compared to other amino acids.

## A STRONGER RMP AMONG INVERTEBRATE MTDNA GENES

**Fig. (3):**
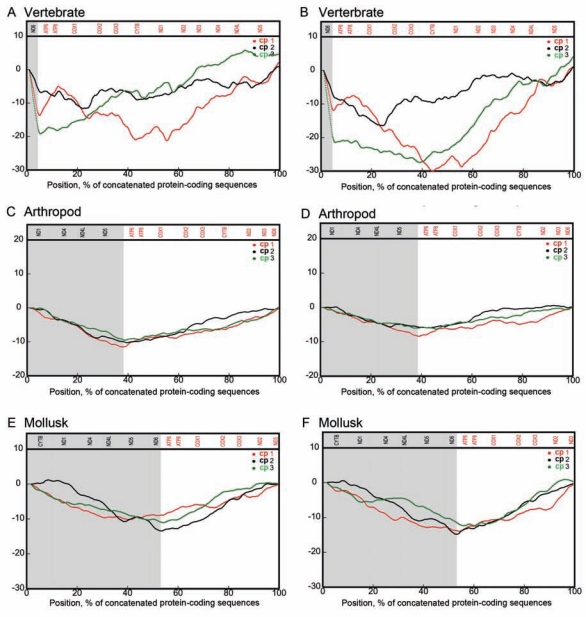
mtDNA composition analysis at the three codon positions based on concatenated 13 mitochondrial protein-coding genes from different taxonomic groups of animals. We used *Chlamydosaurus kingie* and *Cylindrophis ruffus* (**A, B**) as representatives of vertebrates, *Campodea lubbocki* and *Gomphiocephalus hodgsoni* (**C, D**) as representatives of arthropods, and *Vampyroteuthis infernalis* and *Watasenia scintillans* (**E, F**) as representatives of mollusks. Numbers on the y-axis indicate relative nucleotide abundance. The H- or L-strand genes are divided by shading background. Genes marked in red (top) and black are sense transcribed genes of L-strand and H-strand, respectively.

We selected several complete mitochondrial genomes from different taxonomic groups of animals to validate the different trends found between vertebrates and non-vertebrates. Among the vertebrate mtDNA genes, SCA is more pronounced at cp1 and cp3 but weaker at cp2 (Fig. **3A**, **3B**). Such codon-position-specific effects reflect the organization of the genetic code [39-42]. There are several mechanisms may be at work. First, mutational pressure pushes for a compositional change on a strand, altering DNA sequences. The cp1 and cp3 nucleotides are changing more easily under pressures so that we can measure the presence of such pressure. Second, once the amino acids are fixed due to functional selections, purifying selection conveys resistance to further changes, especially for those cp3 nucleotides to avoid loss-of-function mutations (such as at the two-fold degenerate sites). Since the protein-coding nucleotide sequences at cp2 very much determine the properties of amino acids (such as hydrophobicity), amino acids at this position are largely negatively selected in most cases. Third, what is striking about such observations is the fact that, in the case of invertebrate mtDNA genes, RMP appears to show an equal effect on the three codon positions. In other words, codon positions, cp1, cp3, and even cp2 all show similar yet stronger SCA (Fig. **3C** to **3F**). This phenomenon appears universal among most, if not all, invertebrate mitochondrial genomes. This is believed to be a result of balanced mutation and selection [43]. It can be achieved in two ways. First, positive selection and advantageous mutations are so strong that they gradually become fixed at cp2 and the rest sites are left to draft back and forth. Second, relaxed selection or drafting may occur in invertebrate mtDNAs, where the codon bias of all positions reaches an equilibrium [44].

## GENE ORGANIZATION VARIATION AMONG ANIMAL MITOCHONDRIAL GENOMES

Gene organization among animal mitochondrial genomes is highly conserved and related to nucleotide composition variation through the change of codon and amino acid usages. Usually, strand-biased mutations lead to SGD because genes or CDS (coding sequences) favor higher purine contents. The reason lies in the organization of the genetic code where abundant amino acids are in general purine-rich. For instance, two abundant acidic amino acids are both encoded by GAN [35, 39]. However, SGD in prokaryotic genomes are complicated due to frequent horizontal gene transfers. In *Chlamydia* genomes, gene strand-switching decreases as nucleotide composition becomes more biased and most substitution types are asymmetric substitution due to replication-associated mechanisms [45]. In *Bacillaceae* group, horizontally transferred genes prefer the leading strand, and conserved genes are subjected to be discarded more than lineage-specific genes from the leading strand [46]. In vertebrate mitochondrial genomes, recombination is rather rare but it seems not true among metazoan species of the early animal branches according to an analysis on co-cluster genes; recombination occurs in some animal species [22]. A recent study on mitochondrial genome of Antarctic notothenioid showed that its *nad6* gene jumps from H-strand to L-strand through a duplication-loss model [33]. This provides a solid incidence that gene shift occurs under mutational pressure or due to SCA. 

## CONCLUSION

The mitochondrial genome provides a simple model system for study RMP and SCA as well as their relationship to codon and amino acid usages and gene organization. Among animal mitochondrial genomes, SCA of the two differently replicated strands may be introduced by replication-associated mutational pressure [25, 32, 47] and our review suggested this asymmetry is directly related to mitochondrial protein-coding genes since genes on both DNA strands have distinct codon and amino acid usages. The composition and usage variations may also govern the position change of mitochondrial genes of the DNA strands. These statements are consistent with previous studies on bacterial genomes where replication-associated mutational pressure drives codon and amino acid usage changes in protein-coding genes.

Natural selection also acts on codon and amino acid usages because some of these changes alter protein function or biological processes [48-50]. Furthermore, natural selection could also call upon appropriate gene organization in the two DNA strands that often have distinct nucleotide, codon, and amino acid compositions. Therefore, selection can act to promote SGD directly. 

## Figures and Tables

**Table 1.** The Average Relative Synonymous Codon Usage (RSCU) of L- and H-Strand Genes Among Vertebrates and Hexapod mtDNAs

		Endothermic animal^b^		Exothermic animal^c^				Hexapod^d^	
Amino acid (anticodon)^a^	Codon	RSCU (L-strand)	RSCU (H-strand)	RSCU (L-strand)	RSCU (H-strand)	Amino acid (anticodon)	Codon	RSCU (L-strand)	RSCU (H-strand)
Leu(UAG)	TTG	0.09	2.32	0.16	1.84	Leu (UAG/UAA)	TTG	0.24	0.97
	CTT	0.75	0.56	1.23	0.88		CTT	0.75	0.44
	CTA	**2.74**	0.27	**1.93**	0.41		CTA	1	0.22
	CTG	0.34	0.43	0.44	0.46		CTG	0.09	0.07
	TTA	0.98	**2.37**	1.12	**2.28**		TTA	**3.76**	**4.28**
	CTC	1.1	0.05	1.12	0.12		CTC	0.16	0.03
Lys (UUU)	AAA	**1.82**	0.67	**1.73**	0.73	Lys (UUU/CUU)	AAA	**1.75**	**1.37**
	AAG	0.18	**1.33**	0.27	**1.27**		AAG	0.25	0.63
Glu (UUC)	GAA	**1.71**	0.57	**1.61**	0.56	Glu (UUC)	GAA	**1.84**	**1.44**
	GAG	0.29	**1.43**	0.39	**1.44**		GAG	0.16	0.56
Gln (UUG)	CAG	0.19	**1.34**	0.3	**1.25**	Gln (UUG)	CAG	0.11	0.54
	CAA	**1.81**	0.66	**1.7**	0.75		CAA	**1.89**	**1.46**
His (GUG)	CAT	0.63	**1.82**	0.61	**1.56**	His (GUG)	CAT	**1.35**	**1.79**
	CAC	**1.37**	0.18	**1.39**	0.44		CAC	0.65	0.21
Asn (GUU)	AAC	**1.34**	0.16	**1.25**	0.48	Asn (GUU)	AAC	0.45	0.14
	AAT	0.66	**1.84**	0.75	**1.52**		AAT	**1.55**	**1.86**
Ser(GCU/UGA)	TCC	1.61	0.19	**1.54**	0.41	Ser (GCU/UGA)	TCC	0.56	0.15
	TCA	**2.23**	0.59	**1.95**	0.83		AGA	1.37	1.97
	AGC	0.87	0.23	0.97	0.31		TCA	**2.87**	1.32
	TCG	0.14	0.51	0.19	0.61		AGC	0.19	0.12
	TCT	0.91	**2.25**	1.07	**2.61**		TCG	0.14	0.15
	AGT	0.25	2.23	0.28	1.23		TCT	2.09	**2.91**
Trp (UCA)	TGG	0.17	**1.12**	0.28	**1.07**		AGG	0.12	0.18
	TGA	**1.83**	0.88	**1.72**	0.93		AGT	0.65	1.2
Ala (UGC)	GCG	0.08	0.92	0.14	0.87	Trp (UCA)	TGG	0.18	0.43
	GCT	0.77	**2.08**	0.73	**1.88**		TGA	**1.82**	**1.57**
	GCC	**1.71**	0.28	**1.76**	0.41	Ala (UGC)	GCG	0.07	0.21
	GCA	1.43	0.72	1.37	0.84		GCT	**1.69**	**2.69**
Arg (UCG)	CGG	0.18	**1.73**	0.39	**1.56**		GCC	0.61	0.23
	CGA	**2.5**	0.53	**2.37**	0.85		GCA	1.62	0.87
	CGT	0.41	1.66	0.46	1.44	Arg (UCG)	CGG	0.26	0.55
	CGC	0.91	0.08	0.78	0.15		CGA	**2.87**	1.49
Cys (GCA)	TGC	**1.43**	0.25	**1.35**	0.26		CGT	0.67	**1.88**
	TGT	0.57	**1.75**	0.65	**1.74**		CGC	0.2	0.07
Gly (UCC)	GGC	1.28	0.14	1.25	0.27	Cys (GCA)	TGC	0.52	0.12
	GGG	0.36	**1.68**	0.62	**2.07**		TGT	**1.48**	**1.88**
	GGA	**1.82**	0.68	**1.59**	0.63	Gly (UCC)	GGC	0.19	0.15
	GGT	0.54	1.5	0.53	1.03		GGG	0.45	0.91
Asp (GUC)	GAT	0.67	**1.84**	0.64	**1.66**		GGA	**2.52**	1.3
	GAC	**1.33**	0.16	**1.36**	0.34		GGT	0.84	**1.64**
Phe (GAA)	TTT	0.75	**1.87**	0.98	**1.75**	Asp (GUC)	GAT	**1.46**	**1.84**
	TTC	**1.25**	0.13	**1.02**	0.25		GAC	0.54	0.16
Met (CAU)	ATA	**1.67**	0.67	**1.43**	0.74	Phe (GAA)	TTT	**1.57**	**1.91**
	ATG	0.33	**1.33**	0.57	**1.26**		TTC	0.43	0.09
Tyr (GUA)	TAC	**1.17**	0.27	**1.14**	0.39	Met (CAU)	ATA	**1.8**	**1.61**
	TAT	0.83	**1.73**	0.86	**1.61**		ATG	0.2	0.39
Val (UAC)	GTT	0.7	**1.82**	1.05	**1.62**	Tyr (GUA)	TAC	0.54	0.15
	GTC	1.02	0.14	0.97	0.21		TAT	**1.46**	**1.85**
	GTG	0.28	1.22	0.37	1.28	Val (UAC)	GTT	1.48	**2.34**
	GTA	**2**	0.82	**1.61**	0.89		GTC	0.25	0.13
Thr (UGU)	ACT	0.73	**2.31**	0.74	**2.07**		GTG	0.23	0.4
	ACC	1.37	0.15	**1.44**	0.49		GTA	**2.04**	1.14
	ACA	**1.78**	0.87	**1.65**	0.73	Thr (UGU)	ACT	1.47	**2.45**
	ACG	0.12	0.67	0.16	0.71		ACC	0.45	0.26
Pro (UGG)	CCA	**1.69**	0.33	**1.59**	0.6		ACA	**2**	1.13
	CCC	1.36	0.29	1.39	0.54		ACG	0.08	0.16
	CCT	0.85	**3.01**	0.81	**2.07**	Pro (UGG)	CCA	**1.69**	0.86
	CCG	0.1	0.37	0.2	0.79		CCC	0.52	0.27
Ile (GAU)	ATT	0.91	**1.87**	**1.17**	**1.76**		CCT	1.69	**2.69**
	ATC	**1.09**	0.13	0.83	0.24		CCG	0.1	0.17
						Ile (GAU)	ATT	**1.69**	**1.9**
							ATC	0.31	0.1

The **bold** numbers indicate the most frequently used codons.

a What in parentheses are anticodons of the corresponding tRNAs.

b Andothermic animals whose mitochondrial genome sequences are available.

c Exothermic animals whose mitochondrial genome sequences are available.

d Hexapods whose mitochondrial genome sequences are available.
