# Supplementary material for: Replication-Associated Mutational Pressure (RMP) Governs Strand-Biased Compositional Asymmetry (SCA) and Gene Organization in Animal Mitochondrial Genomes
Source: Curr Genomics. 2012 Mar;13(1):28–36. doi: 10.2174/138920212799034811 (PMC3269014; doi:10.2174/138920212799034811)
Supplement: Supplementary file 1 [file CG-13-28_SD1.pdf]

SUPPLEMENTARY MATERIAL

```
tRNA-Ala
(Am)Batrachuperus mustersi        ----AAGGTCTTAGCTTAAT-TAAAGCATCTGGGTTGCATTCAGATAATGTGGGGTAAAATCTTGCAGATCTTA
(Am)Siphonops annulatus           ----AAGGCCTTAGCTTAAAATAAAGTGTCTGAGTTGCAGTTAGAAGATGTTGGATGAAGTCTTGCAGGTCTTA
(Am)Uraeotyphlus cf. oxyurus MW   ----AAGGTCTTATCTTAAT-TAAAGTCCCTGGGTTGCATTCAGGAGATGTTGGATATTATCCTGCAGACCTTA
(Bi)Corvus frugilegus             ----AAAATCCTAGCTTAAT-TAAAGCATCTGAGTTGCATTCAGGGGATGCAGGATAACGTCCTGCGGATTTTA
(Bi)Melopsittacus undulatus       ----AAGGTCCTAGCTTAAT-GAAAGTGTCTGGGTTGCATTCAGGAGATGTAGGTTAGTATCCTACGGATCTTA
(Bi)Menura novaehollandiae        ----AAAATCCTAGCTTAAT-GAAAGCATCTGAGTTGCATTTAGGAGATGCAGGGTAGTATCCTGCGGATTTTA
(Ch)Heptathela hangzhouensis      ------GGTTGGAATT----AATTTTAATTTGAATTGCAATC----AACATTTTGTCTATTTAGACC-------
(Ch)Ixodes holocyclus             ----AGAAAAGTATTTAAATTAATAAAATCTAATTTGCATTTAGAAATTG-GTTTTCCCCTTTTCTA-------
(Ch)Nymphon gracile               --------TAGGTATT----AAATATTTTAAAATTTGCATTTT--TAATAATTCTATTAATTAGAAATATCTAA
(Cr)Megabalanus volcano           -----AAGTAGAAGTTT-TTATA--ATATTTGACCTGCACTCA--GAAGAAGGTAATTTCTATTACCCTACTTT
(Cr)Squilla empusa                ----AGGATAGTAGTC--AAATATGATATCTGATTTGCATTTA--GAAGGTGTTACAAAG-TTAACCTATCTTA
(Cr)Tigriopus californicus        --GAGTTTTAAGTCATTAACTAATGATTTTTTATTTGCACTAA--AAAGGAAATAA--AACTTG----------
(Fi)Anguilla mossambica           ----AAGGCCTTAGCTTAAT-TAAAGCACCTGAGTTGCATTTAGGTTATGTGGGATAAAGCCCTGCAGGTCTTA
(Fi)Diaphus splendidus            ----GAGGCTTTAGCTTAAT-TAAAGTGTCTGTTTTGCATGCAGGAGATGCGGGGTAGAGTCCCGTAAGTCTTA
(Fi)Eleotris acanthopoma          ----AAGGCTTTAGCTTAAT-TAAAGTGTCTGTTTTGCACACAGAAGCTGTGGGTTAGTGTCCCGCAAGTCTTA
(He)Ostrinia furnacalis           ----AGGATTATAGTTTAAATAA-AACATTTGATTTGCACTCA--AAAAATATTGAATAAATCAATTTATCTTA
(He)Periplaneta fuliginosa        ----AGGATAGTAGTT--AAATATAACATTTGGGTTGCATTCA--AAAAGTATTGATTAT-TCAATCTATCTTA
(He)Pyrophorus divergens          TACTAGGATAATAGTTTAAAAAACAACATTTAATTTGCATTTA--AAAAATATTGAATA--TCAATTTAT----
(Ma)Canis lupus familiaris        ----GAGGGCTTAGCTTAAT-TAAAGTGTTTGATTTGCATTCAATTGATGTAAGATAGATTCTTGCAGCCCTTA
(Ma)Didelphis virginiana          ----AAGAATTTAGCTTAAT-TAAAGTATTCGATTTGCGTTCGAGTGATGCAAGATAAAGTCTTGTAATTCTTA
(Ma)Monachus schauinslandi        ----AAGGGCTTAGCTTAAT-TAAAGTGGTTGATTTGCATTCAATTGATGTAAGATAGGGTCTTGCAGTCCTTA
(Mo)Mytilus galloprovincialis     ----AGAATGGTACTTTAAAAAAAAGAAT-TGGTTTGCATCTAATTATTA-AGCCTGAGCTTTCATTCTT----
(Mo)Mytilus trossulus             ----AGAATGGTACTTTAAAAAAAAGGAT-TGGTTTGCATCTAATTATTA-AGCCTAGGTTTTCATTCTT----
(Mo)Watasenia scintillans         ----GGTATTATACTTT-ATATAGAAGATATGATTTGCACTCATAAAGAAAGGTTAATCCTTTGATACCA----
(Re)Cylindrophis ruffus           ----AGAGTTTTAGTTTAAT-TAAAATGTCTGTTCTGCAAGCAGAAGATGTGGG-TAG----CCGCAAGCTCTC
(Re)Manouria emys                 ----AAGGCTTTAGCTTAAT-TAAAGTGCTTGAGTTGCATTCATGAGATATAAGATAAAGTCTTATAGGTCTTA
(Re)Platysternon megacephalum     ----GAGACTTTAGCTTAAT-TAAAGTGTCTGAGTTGCATTCATAAGATATAGGATAGAGCCCTATAGGTCTTA

tRNA-Val
(Am)Bombina bombina               CAAAATGTAGCTTAAT----AAAGCAATTCGCTTACACCGAATAGATGCCCGCTTAAACCG-GACCATTTTGC-
(Am)Ranodon sibiricus             CAAAATATAGCTAAAACA-TA--GCATCTTGCTTACACCAAGAAGATACTTGTTAAATTCA-AGTTATTTTGA-
(Am)Xenopus laevis                CAAAGTATAGCTTAACC---AAAGCCTTTCGCTTACACCGAAACAATATCTGTTAAACCCG-GATTACTTTGA-
(Bi)Ardea novaehollandiae         CAAGGCGTAGCTATAAGA-CAAAGCGCTCAGCTTACACCTGAAAGATATCTACACCCAACA-GATCGCCTTGA-
(Bi)Branta canadensis             CAAGACGTAGCTATAATGCCAAAGCACTCAGCTTACACCTGAGAGATATCT--ACTAAACCAGATCGTCTTGA-
(Bi)Gallus varius                 CAAGGCGTAGCTATAACTTCAAAGCATTCAGCTTACACCTGAAAGATACCCTCAACAGACAAGGTCGCCTTGA-
(Ch)Achelia bituberculata         TAAAAGTAATCTTTA---GATAAGTATATTATTTACAATAATAATT-TCCTGTTAAAATCA-GGACTTTTAA--
(Ch)Carios capensis               TAAGGTGTAAT-CAATA---ATGATATTTCTTTTACA--AGGAAAA-TTAGCTGTGTTTAT-GGCCACTTTAA-
(Ch)Ornithodoros porcinus         TGGAATGAAAT-CACT-----TGATATTTCTTTTACA--AAGAAAA-TAGGCCTTA------AGCCATTCTAA-
(Cr)Daphnia pulex                 AAATAAAGAGCTTGACTAGTTAAGCAACTCGTTTACATCGAGTCTATATTTGTGCAAATCA-AATTTTATTTA-
(Cr)Harpiosquilla harpax          CAAAATAGAGCTTGATTAGTTAAGCACTTCACTTACGTTGAAAAGATATC-GTGCAAATCG-ATTTATTTTGA-
(Fi)Erpetoichthys calabaricus     CAAAATGTAGCTTA-ATAGAAAAGTATCTCCCTTACACTGAGATGAAGTCTGCGCAATTCA-GACCATTTTGA-
(Fi)Percina macrolepida           CAGAGCGTAGCTAAGACAGAAAAGCATCTCCCTTACACTGAGAAGTCCCCCGTGCAAATCG-GAGCGCCCTGAT
(Fi)Satyrichthys amiscus          CAGAGTGTAGCTAAGATAGAAAAGCATCTCCCTTACACTGAGAAGTCACCCGTGCAAGTCG-GGTCACCCTGA-
(He)Aleurochiton aceris           AAATAAATAACTAAACAA-AAAAGTGTGTTATTTACAATAACAAAATGATTTTTAA--AAT-CTTTATTTT---
(He)Drosophila yakuba             CAATTTAAAGCTTATTAAGTAAAGCATTTCATTTACATTGAAAAGATTTTTGTGCAAATCA-ATATAAATTGA-
(He)Ruspolia dubia                CATAATAAAGCTTTAAAAGGTAAGCATTTCATTTACACTGAAAAGAATTTCGTGCAAATCG-AATTTTTTTGA-
(Ma)Cervus nippon yesoensis       CAAGATATAGCTTAAA---CAAAGCACCTAGTTTACGCCTAGAAGATTTC---ACATACCACGAATATCTTGA-
(Ma)Equus caballus                CAAAGTGTAGCTTAAA---CAAAGCATCCAGCTTACACCTAGAAGATTTC---ACTCAAAATGAACACTTTGA-
(Ma)Trachypithecus obscurus       CAAGGTATAGCT-CAATA-CAAAGCATCCTGCTTACACCCGGAAGATATCAACATC--ACTTGATTACCTTGA-
(Mo)Lottia digitalis              TGGGCTATAGTATATTC---TGTATATCTTGCTTACGCCAAGAAAGTCCCCCTTTACCTGG-GGGTAGCCTAA-
(Mo)Octopus vulgaris              CAAAAATTAACATAATTG-TAATGTGTCTCACTTACGTTGAGAGGATAATTTTATT--TAA-ATTATTTTTGA-
(Mo)Watasenia scintillans         CAAGATTTAACATAAAT--TAATGTGTCTCACTTACATTGAGAAAATAATTATAAA--TAA--TTAGTCTTGA-
(Re)Furcifer oustaleti            CAAGAAGTAGCTTA--TT-AAAAGCATTCACCCTACAACTGAAAAATGTT---GATGC-CA--ACCTTTTTGA-
(Re)Testudo marginata             CAAAATATAGCTTAATCTATAAAGCACTCAGCTTACACCTGAAAGACGTCC--ACTATAAACGACTATTTTGA-
```

```
tRNA-Arg
(Am)Amolops tormotus          AAGGTGTTAGTCCAAA--AAAGACAGCTGATTTCGACTCAACTAATTATGGTTTAAGCCCATAGCACCTTT-
(Am)Hyla chinensis            GAGGAGGTAGTCTAAA--AAAGACAGCTGATTTCGACTTAGCAAATTATGGTTCAACCCCATACCCCCTCT-
(Am)Hynobius leechii          --ATATTTAATCTAAA--AAAGACTACTGATTTCGACTCAGTAAATTTTGGTTAAACCCCAAAAATATT---
(Bi)Falco sparverius          AGAAAGTTAGTCTAAT--TAAGACAGTTGATTTCGACTCAACAAACCATAGCCTTATCCTATGACTTTCTT-
(Bi)Gallus varius             AGAAAGTTAGTCTAAC--TAAGACAGCTGGTTTCGACCCAGCAAATTATAGGCCCA-CCTATAACTTTCTT-
(Bi)Phaethon rubricauda       AGAAAGTTAGTCTAAC--TAAGACAGTTGATTTCGACTCAACAAACCATAGCCCGACTCTATGACTTTCTC-
(Ch)Achelia bituberculata     --AAATAAGAAATGATTTATCATAG-TTAATTTCGACTTA-ATTTTAGATT--ATAATATAATCCTTATTTT
(Ch)Nymphon gracile           -----TAACAAGAAATTTTT-------TAATTTCGAACTA-AAACTAGGTT--ATAATAAA-CCCTTGTTAA
(Ch)Ornithoctonus huwena      ------GCAGATAGGTGCTAGGCGGTATGATTTCGGCTCATAATATGGAGATTGAA----------------
(Cr)Harpiosquilla harpax      --AGATAAAAAGCGAAAAATT-GCAATCAGTTTCGACCTGAGGTTTGGTG---TTAATACA-CCTTTATCTG
(Cr)Lysiosquillina maculata   --AGGTAAAAAGCGAAATATC-GCACTCAGTTTCGACCTGACTTTTGGTG---TGAACACA-CCTTTACCTG
(Cr)Panulirus japonicus       --AATTAGAAAGCGAAAATTT-GCTCTTAGTTTCGACCTAAGCCTTGGT----TTAATACA-CCTCTAATT-
(Fi)Alosa alosa               AGGGAATTAGTCCAACT-GAAGACTTCTGATTTCGGCTCAGACAATTGTGGTTAAAGTCCACAACTCCCTT-
(Fi)Bassozetus zenkevitchi    GGGTATTTAGTTTAACA-TAAAACATTTGATTTCGGCTCAAAAGACCGTGGTTTGAATCCACCATTGCCCT-
(Fi)Clupea harengus           AGGGAGTTAGTCCAACT-GAAGACTTCTGGTTTCGGCCCAGAAAATTGTGGTTAAAATCCATAGCACCCTT-
(He)Cydistomyia duplonotata   --GAATATGAAGCGATATATC-GCAATTAGTTTCGACCTA-ATCTTAGA----TGAAGTCA-TCCTTATTCT
(He)Gryllotalpa orientalis    --AAATAAGAAGCAATTATTGCATT--CAGTTTCGACCTG-AAATATGGTG--AAATCA---CCCTTGTTTA
(He)Philaenus spumarius       ---AATAGAGAAGTAATTATA--CAATTAGTTTCGACCTAATAATAAGGAA---ATTTGTTCCTCCTATTT-
(Ma)Equus caballus            TGGTAATTAGTTTAAACCAAAA-CAAATGATTTCGACTCATTAAACTATGATTAA-CTTCATAATTACCAA-
(Ma)Monachus schauinslandi    TGGTAATTAGTTTAACTCAAAAACAAATGATTTCGACTCATTAGACTATGATTTA-CATCATAATTACCAA-
(Ma)Sus scrofa                TGATAATTAGTTTAAAACAAAA-CAAATGATTTCGACTCATTAGACTATGATTTA-CTTCATAATTATCAA-
(Mo)Lampsilis ornata          --AAGCAAGAAA-ACTTCATAGTATTATGATTTCGACCCATAAAT-AGGTA--TACTTATA-CCCTTGCTTT
(Mo)Loligo bleekeri           --CAACAAGAAATGATTAATCATAT-ATGATTTCGGCTCATACATTAGGTG--TACAAACA-CCCTTGTTTA
(Mo)Thais clavigera           --GTATAAAAAGCGAGAAATTTGCATATGGTTTCGGCCCATATCTTGAGGGTGTGAGTCCT-TCTTTATATT
(Re)Furcifer oustaleti        AAGGAATTAGTTTAAA--AAAAACAACAGACTTCGAACCAGTTAACTTAGGTCCA-CCCCTAAATTCCTAA-
(Re)Gekko vittatus            AGATGGTTAGTTTAATA-AAAA-CCACTAATTTCGACTTAGTAAACCCCAAATTAA----GGGACCATCAA-
(Re)Indotestudo elongata      -AATAGCTAGTCCAACAGAAGA-CTACTAACTTCGACTTAGTCAATCGTGATTTAACTTCACGGCTATTAA-
```

```
tRNA-Asn
(Am)Ranodon sibiricus              --TAGATTAAAGCTCGCTGG--ATTGAGCGTTTAGCTGTTAACTAAAAAGTCGTAGGATAAAAGCCTTCTAATCTA
(Am)Rhyacotriton variegatus        --TAGATTAAAGCTCGCTGG--ATGGAGTGTTTAGCTGTTAACTAAAAGGTCGCAGGGTAAAAGCCTGTTAATCTA
(Am)Uraeotyphlus cf. oxyurus MW    --TAGATTGGAGCTCTCTGG--GATGAGCGTTTAGCTGTTAACTAAAAGGCCGTGGGATAAAAGCCCATCAATCTA
(Bi)Egretta eulophotes             --TAGACGGAAGCCTGTTGGG-TTTGGGCATCTAGCTGTTAACTGGAATTTTGCGGGATCGAGGCCCGCCTGTCTA
(Bi)Nisaetus nipalensis            -GTAGGTAGAAGCTCGCTGG--TTGGGGCACCTAGCTGTTAACTAGGGTTTCGTGGGATCAAGGCCCATCTATCTA
(Bi)Pterodroma brevirostris        --TAGACGGAAGCCTGCTGG--CTTGGGCATCTAGCTGTTAACTAGGATTTTATGGGATCGAAGCCCATCTGTCTA
(Ch)Centruroides limpidus          TATTAACAGAAGCT--T-T----TGTAGTGTCCCACTGTTGATGG-GAAGGAG--GG--GAACCCTGTTATGAG--
(Ch)Habronattus oregonensis        -------TAGATAAGGAGCT--GATAGCGTTTTCATTGTTAATGA-AATGAAG--TAGAG----GGGCTGTAACTA
(Ch)Varroa destructor              --TTAATAAAAGCCAAAAT----AGAGGCATTTCATTGTTAATGA-AAATATT--GAAAG--TATTTTTCTTATTA
Clustal Consensus--TTAGCAGAAGCTAAT-T----GATAGCGCTTCATTGTTAATGAAGAAGGAG--GGATGACCCCTGTTAAG------
(Cr)Pseudocarcinus gigas           --TTGAGAGAAGCCAAAATA--TAGAGGCATATCACTGTTAATGA-TAAAATT--GAAA---TTTTTTTCCTGTCA
(Cr)Squilla mantis                 --TTAACCAAAACCAAAGT----TGAGGTATATTATTGTTAATAA-TAGAAAT--GAAAGTTTTTTTTTCTGGTTA
(Fi)Beryx splendens                --TAGATGAATGCTCGCTGG--TTTGGGCGCTTAGCTGTTAACTAAGATCTTGTAGGATCGAGGCCTTCTCATCTA
(Fi)Coilia nasus                   --TAGGCGGATGCTCGCTGG--TTAGAGCGCTTAGCTGTTAACTAAGAGTTTGTAGGATCGAGGCCTTCCTGCCTA
(Fi)Scomberomorus cavalla          --TAGACGGATGCTCGCTGG--TTTGGGCGCTTAGCTGTTAACTAAGAGTTTGTAGGATCGAGGCCTGCCTGTCTA
(He)Homalodisca coagulata          --TTAGTTAAAGCCAAAATT---AGAGGCATCTTATTGTTAATAA-GAATAAT--GAAT-----TTTTTCTAACTA
(He)Pteronarcys princeps           -ATTAATTGAAGCCAAA-T----AGAGGCATATCACTGTTAATGA-TATAATT--GAATA--ACTTATTCCAATTA
(He)Thrips imaginis                --TTAGTTAAAACCAAAAT----AGAGGTATACTACTGTTAATAG-TAAAATT--GAAGA--A--TATTCTAACTA
(Ma)Anomalurus sp. GP-2005         --TAGATTGAAGCCAGTTGT--TTAGGGCGTTTAGCTGTTAACTAAATTTTTATGGGATAAAGGCCCATCGGTCTA
(Ma)Bubalus bubalis                --TAGATTGAAGCCAGTTGATT--AGGGCATTTAGCTGTTAACTAAAGTTTCGTGGGGGTGGAGCCCACCAGTCTA
(Ma)Sorex unguiculatus             --TAGATTGAAGCCAGTTGT--TTAGGGTATTTAGCTGTTAACTAAAATTTCGTGGGATGAAAGCCCACCAATCTA
(Mo)Mizuhopecten yessoensis        --GGAACACTAGTCTATGC----GAGGACGTCCCGCTGTTAACGG-GAAAGAG--GGCGTATAGCTCGTGTTCTT-
(Mo)Placopecten magellanicus       --GAAGCATTAGTCTAT-T----GAGGGCGCCCCGCTGTTAACGG-GGAGGAG--GGGATAAGTTTCCCATGCTTT
(Mo)Pupa strigosa                  --TTGGGTGTAGCTTAAATT--TAAAAGCGTTTAGCTGTTAATTA-AAAGAAT--GACT-----TATGTCTACCCA
(Re)Dinodon semicarinatus          --TAGATTAAAGTCCGCCGG--TTTTGACAGCTAGCTGTTAATTA-GTTTTTGTGGGATCGAGGCCCGCTAATCTA
(Re)Geocalamus acutus              --CGGATTGAAACCCGCTGG--ATTGGGTGTTTAGCTGTTAACTAAGTTCTTGTAGGATCGAGGCCTGCCAATCTA
```

```
tRNA-Asp
(Am)Ambystoma andersoni            -GAGATATTAGTAAAACC----ATTACAATTCCTTGTCAAGGGGCTATTACTAGTTAAAATCTTGTATATCTTTA
(Am)Amolops tormotus               -GAGGGGTTAGTTAA-AC---CATAACGCTGCCTTGTCAAGACAAAATTATTAGTGAAACTCTTGTACCCCTTG-
(Am)Typhlonectes natans            -AGAGTGTTAGTAAAC-T---AATTACACTGCCTTGTCGTGACAACATAATGAGCCTCACCCTCATACACTCTA-
(Bi)Aythya americana               -GAGGCGTTAGTAAACCA----ATTACATAGCCTTGTCAAGGCTAAATCACAGGTGAAAACCCTGTACACCTCA-
(Bi)Ciconia boyciana               -GAGATGTTAGTAAACCA----ATTACGTAGCCTTGTCAAGACTAAATCACAAGTGAAAACCCTGTACATCTCA-
(Bi)Diomedea melanophris           -GGGGCATTAGTAAACCA----ATTACATAGCCTTGTCAAGGCTAAATCACAGGTGAAAACCCTGTACGTCCCG-
(Ch)Carios capensis                --AAAAACTAGT-TAAAAAA-TAACA--TAAGATTGTCATTCTTAAATTACTCCCGTGTTTTTT-----------
(Ch)Ixodes uriae                   --ATGAACTAGT-TAAAAAA-TAACA--TTAGAATGTCATTCTAAAATTACACTGATGTGTTCATT---------
(Cr)Armillifer armillatus          -TGAATTTTAGTTAAACTA--TAACA--TTAACTCGTCAAGTTAAAGTTCATAAATTCAT--------------
(Cr)Hutchinsoniella macracantha    -GAAAAATTAGT-TAATAA--TAATA--TTAGTTTGTCGTACTTAAGTTACTTTT-TGTAAGTATTTTTCG----
(Cr)Portunus trituberculatus       -AAGAAATTAGTTAAACTA--TAATA--TTAACTTGTCATGTTAAAGTCATCTTGTTAAA---GATATTTCTTA-
(Fi)Alepocephalus tenebrosus       -AGGACCCTAGTAAAATAGCACATTACACTGCCTTGTCAAGACAGAATTGTAGGTTAGACCCCTGCGGATCCTG-
(Fi)Caelorinchus kishinouyei       -GAGCTTCTGGTGAAACT----ATCACACTGCCTTGTCGAGGCTGCTTTGTGGGTTAAAGCCCCACGTAGCTTT-
(Fi)Zu cristatus                   -AAGGCATTAGTAAAAAT----ATTACACTACTTTGTCAGGGCAGTAATGCGGGTTAAAATCCCGCATGCCTTA-
(He)Philaenus spumarius            -TAAAAATTAGTTTAATTA--AAATA--TTAATTTGTCAAATTAGAGAATTTGAAATAAT-TCAAAATTTTTA--
(He)Thermobia domestica            -GAAAAATTAGTTAAATTA--TAACG--TAAGCATGTCAAGCTTAAATTACTACC-CAATAGTATTTTTCT----
(He)Tricholepidion gertschi        -AAAAGATTAGTTAAAAAA--TAACA--CAAGTATGTCAAACTTATATTATTAATATAATATTAATATCTTTTA-
(Ma)Bos grunniens                  CGAGGTGTTAGTAAAAC-----ATTATATAACTTTGTCAAAGTTAAGTTACAAGTGAAAACCCTGTACGCCTCA-
(Ma)Phoca fasciata                 -GAGGCATTAGTAAAA------ATTACATAACTTTGTCAAAGTTAAATTATAGGTGAAAACCCTTTATGCTTCC-
(Ma)Rattus norvegicus              -GAGATATTAGTAAAATAA--TTACA--TAACCTTGTCAAGGTTAAGTTATAGACTTAAATCTATATATCTTA--
(Mo)Albinaria coerulea             -TTTTACTTAATTTATAT---TAAAATAATTTCTTGTCATGAAA---TAAATAACAGCAATGTGTTAGTGTAGA-
(Mo)Crassostrea gigas              -AGACAGTTAGTATAGCAAA-TAATACGTTAGTTTGTCGTACTAAAGACACTTTTATGCGAGTACTGTCTT----
(Mo)Watasenia scintillans          -AAGAAGTTAGGTAAACTA--TAACA--TAAGATTGTCAATTTTGAATTACTAATTTA---TTAGTACTTCTTA-
(Re)Boa constrictor                -GGGGGCCTAGTAAATAT---TATTACATGACCTTGTCGTGGT----CAAATAACAGCACTCTGTGGCCCCCA--
(Re)Teratoscincus keyserlingii     -GAGACTCTAGTAAATTA-----TTATATAGCTCTGTCAGTGCTAAGTTATAGG--ACTTCCCTATGAGACTCA-
(Re)Varanus niloticus              -AGGACCCTAGTAAATCA-----TTACATAGCCCCGTCAGAGCTAAATAATAGGCTATACCCCTATGAGTCCTA-

tRNA-Cys
(Am)Ensatina eschscholtzii         --------AGCACCGTAGTGTAAAGAGCACGTAAGATTGCAAATCCTC-CAGAGCCACTT-CTTTTTGGCCGGGGC
(Am)Hydromantes brunus             --------AGTTCTGTGGTGTATA-AGCAAATGAAATTGCAAATTTCA-GAGAGCAGATA-ATTTTCTGTCGGGGC
(Am)Mantella madagascariensis      --------GGCCCTGGGGTGTCA---GCATGTGAAGTTGCAAACTTCA-AGAAGCAGAGT-AATTTCTGCCGGGGC
(Bi)Egretta eulophotes             --------GGCCCTGTGGTGAAGT--TCATGTTGAGTTGCAAGCTCAT-TGATGTACATTAAAAGTGTGCCAGGGT
(Bi)Gallus gallus gallus           --------GACTCTGTAGTGAAGT--TCATAATGAGTTGCAAACTCGT-TGATGTACACTAAA-GTGTGCCGGGGT
(Bi)Numida meleagris               --------AGCCCTGTAGTGAAGT--TCATGGTGAGTTGCAAGCTCAT-TGATGCACATTAAAGATGTGTCAGGGT
(Ch)Amblyomma triguttatum          -----------AATCTTAATTTTTAAAATTT--AAACTGCAAATTTAA-ATGTGAATTATAT-----TTCTAAGAT
(Ch)Leptotrombidium akamushi       --------GATCATCAGTCTTTTGAAAGATATTAAATTGCAAATTTAA-AGA---AAAATTG-----ATCT-----
(Ch)Limulus polyphemus             --------GGCTTTATAGTTTAATTAAAATTTTAAATTGCAAATTTAA-AGATACATTTAGG-----TGTTAGGGC
(Cr)Eriocheir sinensis             --------GGCCCTATAGTATAAAAATAATATTAAATTGCAAGTTTAA-AGATGTGTAAATC------ACTAGGGT
(Cr)Fenneropenaeus chinensis       -GGCTTTGTAGTGGAAATTATAAATAAGACGTCAGACTGCAATTCTGA-AGATG-CGTAGAA-----CGCCGGAGC
(Cr)Ligia oceanica                 ---GGCCCTCATTAACCTTAGTCTGCAAAACTAGGGGTGCAATTACTGCGAGAGTCT-----------------...
(Fi)Phoxinus perenurus mantschu    --------AGCCCCGTAGT-GAAGAATCACGTTGGATTGCAAATCCAG-AGACGCAGAGTAATACCCCGCCGGGGC
(Fi)Pseudobagrus tokiensis         --------AGTCTTGTGGTGTATT--ACACATCGGATTGCAAATCCGA-AGATGCAGGCTAATTGCCTGCCGGGGC
(Fi)Verasper moseri                --------AGCTCCGGGGTGTTA----CATGTTAGATTGCAAATCTAA-AGAGGCAGGCT-AATCCCTGCCGGGGC
(He)Aedes aegypti                  --------AACTTTATATTCAATAAT-GATATTAGACTGCAATTCTAA-AGGAATAATTAAAA-AATTATTAAAGT
(He)Coreana raphaelis              --------AGTCTTATAGTCAAAAAAAGATATAAAATTGCAAATTTTA-AGTTGTATATTAA-----TACTAAGGC
(He)Pyrophorus divergens           --------GGTCTTATAGTCAATAAT-GATTTTGAATTGCAAATTCGA-AGGTGAATGATAA-----TTCTAAGGC
(Ma)Microtus rossiaemeridionali    AGCCTTAGTAGAGTCAGTCCTCT-ACACCTTCGAATTTGCAATTCGAT-GTG---ATTATCA-----CCTTAAGGC
(Ma)Muntiacus reevesi micrurus     --------GGTCCTGTGGTGAAAT-TTCATGTTGAATTGCAAATTCAA-AGAAGCAGCTTCAAACTCTGCCGGGGC
(Ma)Orycteropus afer               --------AGCTCTGAGGTGAAAT-TTCATGTCGAATTGCAAATTCGA-AGAAGCAGCTTCAA-TTCTGCCGGGGC
(Mo)Acanthocardia tuberculata      ---------GGCCAAAAAGCTGACATAGCGGTCTACCTGCAAAGTAGGCTGGGGTAGGATTC-----TACTTTGGT
(Mo)Lottia digitalis               --------ATAGGCGTAGTTTACTAA-AACACTGAACTGCAAACTCAG-AATCACGGGACACTCCGTCGCCTATT-
(Mo)Mizuhopecten yessoensis        --------AGCTCTGTAGTTGAAGAACAACAGTAAGCTGCAGTCTTGCTAGTGGTGGATTATGAACCCCCAGAGC
(Re)Acrochordus granulatus         ------------------AGCCCCGATTTTGCCAGATTGCAAATCCGG-AAAAGGCGAATTT-----GCCCGGGGC
(Re)Coleonyx variegatus            --------AGCCTCGAGGTGAATT---TACGCCAAAGTGCAAATTTGG-AGACGCACCCTAAAAGG-TGCCGGGGC
(Re)Dinodon semicarinatus          ----------AACCCGGAGGTGTGTAACATGTCAGACTGCAAATCTGA-AGTCGGC--AGCG-----GCCCCGGGT
```

```
tRNA-Gln
(Ch)Ixodes hexagonus               ----AGTTTTTGGTGT-AT----ATGCACAATAAATTTTGATTTTATAAGAAATAATTTTG-AATTATTAAAACTA
(Ch)Ixodes persulcatus             ---TAATTTTAAGTGTTAT----TTGCACGTTAAATTTTGAATTTAAAAGAAATAAATTAA-TTTTATTAAAATTA
(He)Nesomachilis australica        ---TGTGTTTTGGTGTATG----GTGCACATTATATTTTGATTATATAGGTAGCAGTTTAA-TTCTGTTAAATGCA
(He)Culicoides arakawae            ---TATATTTTAGTGTACG----AAGCACAAAAGATTTTGATTTTTTTAGATACAGTTTAA-TTCTGTTAAATATA
(He)Homalodisca coagulata          ---TGTAAATTAGTGTATG----ATGCACATAAATTTTTGATATTTAAAGATA-AGTTTAA-TTCT--TAAATTTA
(Am)Desmognathus wrighti           ---TAAAAAATAGTGTAGT--GGGTGCACTTGAAGTTTTGATCTTTAGGGGATAGGTTCAAGTCCTATTTT-TTTA
(Am)Plethodon petraeus             ---TAAGAAGTGGTGTAGT--GGATGCATTTAAAGTTTTGAGCTTTAAGGGGCGGGTTCGAATCCTGAGTT-CTTA
(Bi)Gallus gallus gallus           ---TAGAAAATAATATAGA--GGGAGTATGAAGAGTTTTGATCTCTTCTGTGTAGGTTCGATTCCTACTTT-TCTA
(Bi)Phaethon rubricauda            ---TAGGAAATAATATAGG--GGAAGTATGGGGAGTTTTGATCTCCTCTGTGCAGGTTCGACTCCTGCTTT-TCTA
(Re)Dogania subplana               ---TAGAATGTAGTATAGT--GGAAGTATGAAGAGTTTTGATCTCTTAGGTGTAGGTTCAAATCCTATTTT-TCTA
(Re)Chlamydosaurus kingii          ATGTAGAGAATAGTATAGGT-GGGAGTACTAAAAATTTTGAATTTTTAAATG-GCCTTCGAATCCTCTTTC-TCTA
(Re)Pantherophis slowinskii        ---TAGGAAGTAGTATATT--GGTAGTATAGAAAGTTTTGGGCTTTCTAGTCTAAGTTCGAGTCTTAGCTT-TCTA
(Fi)Lamprogrammus niger            ---TAGAAAGTGGTGTAGT--GGAAGCACCAAGAGTTTTGATCTCTTAAGGATGGGTTCAAGTCCCTTTTT-TCTA
(Fi)Pseudolabrus sieboldi          ---TAGGAAGTGGTGTAGT--GGAAGCACTAAGAGTTTTGATCTCTTAAGGTGGGGTTCGAGTCCCCTCTT-TCTA
(Ma)Microtus rossiaemeridionali    --TTAGAATGGGGTGTAAT-GGGTAGCACGGAGAATTTTGGATTCTCAGGTATAGGTTCGATTCCTATTGT-TCTA
(Ma)Monodelphis domestica          ----AGAATGTGGTGTAA--AGGAAACACGTGGAATTTTGAGTTCTTAAATGTGGGTTCAACTCCTACTGT-TCTA
(Ma)Hemiechinus auritus            ---TAGGGTATAGTGAAATTAGGTAGCACGAAGAAATTTGGATTCTTATGAGTAGGTTCGACTCCTATTAC-TCTA
(Cr)Gonodactylus chiragra          ---TATACTCTAGTGTAGT----G-GCACATTAGATTTTGATTCTTGAGGTAATGGGGCTA-TCCCATTGAGTATA
(Cr)Penaeus monodon                ---TATACTCTGGTGTATA----GTGCACACAAAGTTTTGATCTTTGGGGTAATGGTGTTAATTCCATTGGGTATA
(Cr)Pagurus longicarpus            ---TATATGTTAGTGTAAA----T-GCATAAGAAGCTTTGACCTTCTAGGTTTTGGTGAGAATCCT-TTACATATA
(Mo)Biomphalaria glabrata          TCCTTCCCTTCAGTGTTTG------GCACGAGAAAATTTGAGTTTCTTAGA---GATTTTTTCCAAGGGAAAA---
(Mo)Roboastra europaea             ---ATCTTTTTGGTGTAAT------GCACGGGAGTTTTTGATTCTCCAGGA---GGGTTAGACCCAGAAGATT---

tRNA-Glu
(Am)Bolitoglossa n. sp. RLM200     -TTTCTTATAGTTGAAT-AC----AACGGTGATTTTTCAGATCACAGGTCTCAGAGTG-GTCTGGGTAAGAATT--
(Am)Microhyla okinavensis          -GTTCTTGTAGTTGAATTAC----AACAATAGTTTTTCAGATTATTAGTCCAGGTTGAAGTCCTGGTAGGAATA--
(Am)Thorius n. sp. RLM2004         ATTTCCCGTAGTTGAAT-AC----AACGGCGGTTTTTCATGTCGCTGGTCCCAGTTTA-TTCTGGGCGGGAATA--
(Bi)Menura novaehollandiae         -GTTCCTGTAGTTGA-ATTCTAGTAACGGTGGTTTTTCAGGCCGCAGATCTTGGAGAAAAGCCAAGTAGGAACC--
(Bi)Ninox novaeseelandiae          -GTTTCTATAGTTAAGAGTTTATTAACGGCAGCTTTTCAGGCTGCAAACCTTGGATAGAGGCCAAGTAGGAACT--
(Bi)Podiceps cristatus             -GTTCCTATAGTTGAGAGTC----AACGATGGCTTTTCAAGCCATAGGTCTTGGAGAAAAGCCAAGTGGGAACTG-
(Ch)Heptathela hangzhouensis       -GTTTTTGTAGTGTAA-TTAC----ACATGTTCCTTTCATGAACAAAATAGAATTTCTCAAAAATA----------
(Ch)Leptotrombidium deliense       -ATCTCTTTAGTCTTT-----------AATTGATTTTCGAGTTTCATTCGAAAGAACCAAAATTTGGAAGAGAAA-
(Cr)Lepeophtheirus salmonis        -ATTACTA-GGCTAAGCCTC------CTTTACAGCTTCAATGTAATACTCTTTAT--AAGTTATAGTAATT-----
(Cr)Triops cancriformis            -ATTGTTATAGTTTAA-AAAA---AACTTTACATTTTCATTGTGAAAATAGGTAAACTTACCTTAACGATA-----
(Cr)Vargula hilgendorfii           -CTTAATATAGTATAA-TTAT---TACATTATCCTTTCACGATAAAAAGGGGTAATTTCTACCCTATTAAGA----
(Fi)Carangoides armatus            -GTTCTTGTAGTTGAATAAC----AACGGTGGTTTTTCAAGCCATTAGTCCTGGTTAAAGTCCTGGCAGGAATT--
(Fi)Pagrus auriga                  -GTTCCTGTAGTTGAGTGAC----AACGGTGGTTTTTCAAGCCATGTGGCCGGGTTAAAGTCCCGGGAGGAATT--
(Fi)Phenacobius mirabilis          -GTTCCTGTAGTTGAATTAC----AACGATGTTTCTTCAAGTCATTAGTCTCAGTTAAAGTCTGAGCAGGAATT--
(He)Coreana raphaelis              -ATTTATATAGTTTAATA-AA---AACTTTACATTTTCATTGTAATAATAAAAAAAT-TTTTTTATAAATA-----
(He)Ostrinia furnacalis            -ATTTATATAGTTTAATATAA---AACATTACATTTTCATTGTAAAAATAAAAAAAAATTTTTTATAAAT------
(He)Trichophthalma punctata        -ATTTATATAGTTTAA-ATAA---AACCTTACTTTTTCATTGTAAAAATAAAGAATT-TCTTTTATAAGTT-----
(Ma)Hippopotamus amphibius         -GTTCTTATAGTTGAATTAC----AACGATGGTTTTTCATGCCGTTGGTTATGGTTAGAGTCCATATGGGAATA--
(Ma)Mus musculus                   -GTTTCTGTAGTTGAATTAC----AACGATGATTTTTCATGTCATTGGTCGCAGTTGAATGCTGTGTAGAAATA--
(Ma)Pan paniscus                   -GTTCTTGTAGTTGAAATAC----AACGATGGTTTTTCATATCATTGGTCGTGGTTGTAGTCCGTGCGAGAATA--
(Mo)Mytilus galloprovincialis      -GCTTAAGTAGTTTAG-GGAA---AACATAAGATTTTCATTCTTAAGTCAGAAAGCAGTTCTCT-TAAGTT-----
(Mo)Pupa strigosa                  -GTCGCGGTAGTATAA-AAAT---A-CGCTACTCTTTCGTGGT-AGAGCAGGCAATCACACGGAGATTGCCCCGTG
(Mo)Sepioteuthis lessoniana        AATTCTTATAGTGTAGAAAG----CACATTAGATTTTCAATCTTTTAGTAC--ACTGAGGTGTTGGGAATA-----
(Re)Chlamydosaurus kingii          -ATTTCTATAGTTGAATAAC----AACGTTGATTTTTCAGATCAAAGGTTCAAGC---AGGTTTGATGGGAATA--
(Re)Leptotyphlops dulcis           -GTTTCTATAGTTGAAATAC----AATAGCGGTTTTTCGGGTCGCGGGTCCCG-------TTTGGGTGGAAACT--
(Re)Varanus niloticus              -GTTTTTATAGTTGAATAAC----AACGTGGATTTTTCGTGTCCTTGGTTTTGGATAG--TCCAAATAAAAATA--
```

```
tRNA-Gly
(Am)Onychodactylus fischeri      -TCTTTTTAGTAAAATA---AT-ACAAATGACTTCCAATCATTAAATTCTAG-TTAAACTCTAGAAAAAGATA---
(Am)Thorius n. sp. RLM-2004      GTCTTTTTAGTATAAAA---AT-ACAAATGACTTCCAATCATTCAATCTTAG-CTG--CCCTAAGAAAAGACA---
(Am)Xenopus laevis               ACTTTCTTAGTATTAA--CCAGTACACGTGACTTCCAATCACAAAGTCTTAG-TTAGAATCTAAGAGAAAGTA---
(Bi)Anser albifrons              GCTCTTCTAGTATATTA---ATTACAATTGACTTCCAATCTCTAGAATCTGG-TATAAACCCAGAGAAGAGCA---
(Bi)Falco sparverius             GCTCCCCTAGTATATTA---ATTACAATTGACTTCCAATCTCTAAAATCTGG-TATAACCCCAGAGGAGAGCA---
(Bi)Gallus lafayettei            -CTCTTCTAGTATACTC---ATTACAACTGACTTCCAATCTTTAAAATCTGG-TACCAACCCAGAGAAGAGCA---
(Ch)Ixodes uriae                 ACTACATTAGTATTAAT-AAGGTACATATAATTTCCAATT--ATAAAGTCAA-----ATTTGATGTAGTA------
(Ch)Limulus polyphemus           ATCTTTTTAGTAT--AA-AAAGTACATTTAGCTTCCAACTAAAAAGTTTAAA------ACCTTAAAAAAGATA---
(Ch)Nephila clavata              GATTTATTAGTAT--AT-AAG-TACATTTAATTTCCAATTAAATAGAGAGAA-----AATTTTTTTAAGAATTT--
(Cr)Argulus americanus           ATTTTTTTAGTATGAA----AGTATAACTAACTTCCAATTAGTAGGTCCT--------ATATAGGAAAAAATA---
(Cr)Tigriopus japonicus          --TATTGTAGTATAATT-TAT-TATATTTAGTTTCCACCT--AAAAGATTGC-----ATCAATAATAGGGG-----
(Cr)Vargula hilgendorfii         GTTTGTTTAGTAT--AA-GTAGTATGTTTGATTTCCAATCAAAAGGTTTAAA-----TAGTTTAAAACAAATA---
(Fi)Arcos sp. KU-149             ATTTTTCTAGTACTAAAGTTAGTATAAGTGACTTCCAATCACAGGGTCTTGG-TTAAACTCCAAGGAAAAATA---
(Fi)Eigenmannia sp.              ATCTTTCTAGTATTAAA-T-AGTACAAGTGCCTTCCAATTACTCAGTCTTGG-TTAAAGTCCAAGGAAAGATA---
(Fi)Rhodeus uyekii               ATCTTTCTAGTATTAAA-TTAGTACAAGTGACTTCCAATCATTTAGTCTTGG-TTAAACCCCAAGGAAAGATA---
(He)Anoplophora glabripennis     ATTTATATAATAT--AA-TAATTATATTTGACTTCCAATCAAAAAATCTAGA-----CAACTAGATTTTAATT---
(He)Thermobia domestica          GCCTATTTAATAT--AT-ATAGTATATTTGACTTCCAATCAAAAAGATCA-----------TTAAATGAAATAGGC
(He)Trialeurodes vaporariorum    TTTCTATTAGTATAATA-TAAGTACACTTGATTTCCAATT--AAGGGGTTGT-----GGGTACAATAGAATT----
(Ma)Pteropus scapulatus          ACCCTTTTAGTATAAAT---AGTACAATTGACTTCCAATCAATTAGCTTCGG-TCATAATCCGAAAAAGAGTA---
(Ma)Sciurus vulgaris             ACTTCCCTAGTATCAA--TTAGTACAGCTGACTTCCAATCATCCAGTCCTAG-T-ATAATCTAGGGGGAAGTA---
(Ma)Sorex unguiculatus           ATTCTTCTAGTATTAA--CTAGTACAACTGACTTCCAATCAGTTAGTCTCAG-TATAAATCTGAGGGAGAATA---
(Mo)Dosidicus gigas              ATTTTATTAGTAT--AA-ATAGTATGCTTGTTTTCCAAACAAGTGGTTTAAG-----ATTCTTAAATAAAATA---
(Mo)Lampsilis ornata             ATGCTGAAAGNATATT----AGTACAACTGCCTTCCAAGCAGGAAGCCCAAA-------ATTAGGTCAGCATA---
(Re)Macroclemys temminckii       GCTCCCCTAGTATAACA---GT-ACAAGTGACTTCCAATCACTAAGTTTTAG-TTTAATCCTAAAGAAGAGCA---
(Re)Ovophis okinavensis          ATTTCTTTAGTATACA----AGTATAAATGCCTTCCAAGCATGAGGCCCC--------CCCCGGGAAGAAATA---
(Re)Testudo graeca               ACTTTTCTAGTATAATA---GT-ACAAGTGACTTCCAATCACTAAGTTTTAGCTCTAGCCCTAAAGAAAAGTA---


tRNA-His
(Am)Batrachuperus pinchonii      GTTTAAATAATTTAA-TT---AAAGTACTAGATTGTGATTCTAGAAATAAGAGTTAAACTCTCTTTTTAAACC
(Am)Discoglossus galganoi        GTAGGCATAGTTCAA-CC---AGAATTTTAGATTGTGATTCTAAAGATAGAAGTTAAAATCTTCTTGCCAACC
(Am)Limnonectes fujianensis      GTGAATATAGTTT-AGAC---AAAACCCTAGATTGTGATTCTAGAAATGGGGGATAAAATCTCCCTATTCACC
(Bi)Branta canadensis            GCAAGTATAGTTTAA-AC---CAAACATTAGACTGTGATTCTAAGAATAGAAGTTCAAACCTTCTTACCTGCC
(Bi)Cygnus columbianus           GCAAGTATAGTTTAA-AC---CAAACATTAGATTGTGATTCTAAAAATAGAAGTTCAAACCTTCTTACCTGCC
(Bi)Melopsittacus undulatus      GCAAGTATAGTTTAA-AC---CAAACATTAGACCGTGACCCTAAAAATAGAAGTTAAACCCTTCTTACCTGCC
(Ch)Haemaphysalis flava          GTTTAATTAGTTTAAATTTTAAAAACAACAAATTGTGGATTTGTGGATATGTAAATATTAAACA---------
(Ch)Heptathela hangzhouensis     TTCTTTATAGTTAAA-TT---ATAATATCAGATTGTGGTGCTGA-----AGTTTATTAGAGAAAAT-------
(Ch)Leptotrombidium pallidum     CTCTTTATAGTTTAA-TA---AAAATATCAAATTGTGGTTTTGA-----AGATTTTTAGGAGA---------
(Cr)Cherax destructor            ATTTAAATAGTTTTAATT--AAAATT-TTGGTTTGTGGTACCAAAGATATAAAAAAGTTTTATTTTAAATC--
(Cr)Pagurus longicarpus          GTTCAAATAGTTTAAATA--AAAAACGTTGGTTTGTGGTACCTAAGATATAATT--GTGTTATTTTGAATC--
(Cr)Tetraclita japonica          GTTCAAATAGTTTAAGAG--AAAATT-TAAGTTTGTGGTACTTGAGATGTAA----GGTTTACTTTGGACT--
(Fi)Halichoeres melanurus        GTGGACATAGTTTAA-GC---AAGACAACGGGTTGTGATTCCAGAGATAGGGGTTAAAGTCCCCTTGTCTACC
(Fi)Pellonula leonensis          GCAGATATAGTTTAA-CA---AAAATGTTGGATTGTGATTCCAAAGACAGGGGTTCAAACCCCCTTATCCGCC
(Fi)Triacanthus biaculeatus      GTAGATATAGTTTAA-AC---AAAACGCCAGACTGTGACTCTGGTAACAGAGGTTAAAATCCTCTTATCCACA
(He)Japyx solifugus              ATCTCAGTAGTTTAA-TT---AGAATGTTAACTTGTGGCGTTAATGGTGAGTTTTCTTGGGGTA---------
(He)Onychiurus orientalis        -TTTAGATAGTTTATAA---AAAATA-GAAGATTGTGGGGCTTCAGATG-------GGTTTCCTCTAGAT---
(He)Tetraleurodes acaciae        ATTTGGATAGTTTAA-TA---AAAACATTAATCTGTGTAATTAAAAATAATAATATTTCCAAATT--------
(Ma)Artibeus jamaicensis         GTAAGCATAGTTTAA-AC---AAAACATTAGATTGTGAATCTAAAAACAGAAACTTAATCTTTCTTGCTTACC
(Ma)Pongo pygmaeus               GTAAATATAGTTTAAC-C---AAAACATTAGATTGTGAATCTAATAATAGGGCCCCACAACCCCTTATTTACC
(Ma)Pontoporia blainvillei       GTAGATGTAGTTTAA-AA---AGAACACTAGTTTGTGAAGCTAGCAACAGAAGATAAAACCTTCTTATCTACC
(Mo)Lottia digitalis             GTGGGGCTAGTTTAT-TA---AAAATGTAGGTCTGTGGAACCTAAGTAAGTGTGATTGCACTGCCCGACT---
(Mo)Sepia officinalis            ATAAAGTTAGTTTAAATT---AAAACGATAAGTTGTGGTCTTATAAATAAATAAATAATTTACTTTATT----
(Mo)Venerupis  Ruditapes  phili  TCTAGTTTAGTTTATATT---AAAATGTTGTTTTGTGGTAGCAAAGAAA-----ATTATGTTAACTGGTT---
(Re)Chinemys reevesi             --TAATATAGTTTCAAAA---CAAACGTTAGACTGTGGCTCTAAAAATAGGAGTTAAAATCTCCTTATAAACC
(Re)Gekko vittatus               GTAAGCATAGTTTAATAC---AAAACACTAAGCTGTGGACTTAGTAATAGGGCTTTACA-CCCCTTACATACC
(Re)Pogona vitticepes            GCCAAGATAGTTAAA-AA---ATAACATTAGGTCGTGACCCTAAAGATAGAGGTTTGTGCCT-CTTCCCGGCC
```

```
tRNA-Ile
(Am)Pachyhynobius shangchengens    AGGACATGTGCCCGAAAGT-TAGGACTCACTTTGATAAAGTGATATATAGGGGTTCA--AACCCCCTCATTTCCTT
(Am)Plethodon elongatus            -GGATATGTGCCCGAAAAC-TAAGGCTCACCTTGATAGGGTGAATTATAGAGGTTCA--AATCCCCTCATATCCT-
(Am)Rhyacotriton variegatus        -GGATATGTGCCCGAAAA--TAGGGTTCACTTTGATAGGGTGATATATAGAGGTTAA--AATCCTCTCCTCTCCT-
(Bi)Coturnix japonica              -GGAAGCGTGCCTGAACA--AAAGGGCCACTATGATAAAGTGGAC-ATAGAGGTAAAACAACCCTCTCGCCTCCT-
(Bi)Larus dominicanus              -GGAAATGTGCCTGAACGT-AAAGGGTCACTATGATAAAGTGAAC-ATAGAGGTATACCAGCCCTCTCATTTCCT-
(Bi)Synthliboramphus antiquus      -GGAAATGTGCCTGAACGTTAAAGGGTCACTATGATAAAGTGAAC-ATAGAGGTATACCAGCCCTCTCATTTCCT-
(Ch)Habronattus oregonensis        -ATTAAGGTGCCTGAC-T--AAAGGGTTAATTTGATAGATTAA---ATAAAGTTAGATT----------------
(Ch)Ornithodoros porcinus          -AATAAAATGCCTGA----AAAAGGGGTATCCTGATAGGATAATTCATGTATAA-TTATACTTTTATTA-------
(Cr)Artemia franciscana            -GATGGGGTGCCTGATAA--AAAGGATTACGTTGATACTGTAATT-ATGTAGATAAT--ACCCCCATCT-------
(Cr)Megabalanus volcano            -AATAAGGTGCCTGAT-A--AAAGGGTTACTTTGATAGAGTAA---ATCATGTAAGGCCAGACTTACCCCTATTA-
(Cr)Pseudocarcinus gigas           -AATATAGTGCCTGATTTTAAAAGGATAGCTTTGATAGAGCTAATCATGTATT--TTATACCTATATTA-------
(Fi)Amia calva                     -GGAATCGTGCCCGAACGCCAAAGGACCACTTTGATAGAGTGTATTATGGGGGTTAA--AGTCCCCTCGCTTCCT-
(Fi)Salarias fasciatus             -GGAGCTGTGCCTGAACT--AAAGGGCTACTTTGATAGAGTAGACTATGAGGGTTAA--AGCCCCTCCAACTCCT-
(Fi)Zenion japonicum               -GGAGCTGTGCCTGAATT--ATAGGACCACTTTGATAGAGTGAAACATGTGGGATAA--AGACCCTCCAGCCCCT-
(He)Anopheles quadrimaculatus A    -AATGAATTGCCTGATA--AAAAGGATTACCTTGATAGGGTAAATCATGAAATT-TAATATTTCATTC--------
(He)Heterodoxus macropus           -TACAAAGTGCCTGATTA--AAAGGGTTATTTTGATAAGATAAGC-ATAGTACTAAATTAGTACTCTTTGTAA---
(Ma)Chlorocebus aethiops           -AGAAATATGTCTGACAA---AAGAGTTACTTTGATAGAGTAAATGATAGGGGCTCC--AACCCTCTTATTTCTA-
(Ma)Phoca sibirica                 -AGAAATATGTCTGACAA---AAGAGTTACTTTGATAGAGTAAATAATAGAGGTTTA--AACCCTCTTATTTCTA-
(Ma)Trachypithecus obscurus        -AGAAATATGTCTGACAA---AAGAGTTACTTTGATAGAGTAAATAATAGAGGTGCTT-AATCCTCTTATTTCTA-
(Mo)Albinaria coerulea             -AAGCAGCCGCCGGAATT-GTACGGGTATCATTGATGTTGATAAATATGGAGTTGAATACCGTTGCTTA-------
(Mo)Hiatella arctica               -GGTACTGTGCCAGA--A--AGTGGGTTACTTTGATGTGGTAAAA-ATAAGAGGGAGTCTCTTCGGTATCT-----
(Mo)Nautilus macromphalus          -GATATTGAGCCGGAAT---AACGGATTACATTGATGTTGTAAATCACGGACATATTATGTACCCAATATCC----
(Re)Amphisbaena schmidti           -GGACGCGTGCCTGAATCTAAAAGGGTTACTTTGATAGAGTAAACTATAGGGGCCCC--ATCCCCCTCGCTTCCT-
(Re)Chelonia mydas                 -GGACACGTGCCTGAACA---AAGGATCACCTTGATAGGGTGAATAATAGAGGTTAA--AATCCCCTCGTCTCCT-
(Re)Pogona vitticepes              -GGAAACGTGCCTGAAAC-ATAAGGACTACTTTGATAAAGCAGAC-ATGGAGCCATA--AACTCCC--GCTTCCC-

tRNA-Leu
(Am)Batrachuperus gorganensis      -ACTTTTAAAGGATAATAGTAA-TCCATT-GGTTTTAGGAACCAAAAACTCTTGGTGCA-ACCCCATGTAAAAGTT
(Am)Batrachuperus mustersi         -ACTTTTAAAGGATAATAGTAA-TCCATT-GGTCTTAGGAACCAAAAACTCTTGGTGCA-ACCCCATGTAGAAGTT
(Bi)Falco sparverius               -GCTTTTAAAGGATAGTAGAAA-TCCACT-GGTCTTAGGAACCAATCACC-TTGGTGCA-AATCCAAGTAAAAGCA
(Bi)Gallus varius                  -ACTTTTAAAGGATAAGAGCAA-TCCGTT-GGTCTTAGGAACCACCCATC-TTGGTGCA-AATCCAAGTAAAAGTA
(Bi)Gavia pacifica                 -ACTTTTAAAGGATAACAGTAA-TCCACT-GGTCTTAGGAACCACTCATC-TTGGTGCA-ACTCCAAGTAAAAGTA
(Ch)Ixodes hexagonus               -ACTAATTTGGCAGAAAAAT---TGTGTC-AAATTTAGAATTTGAGTA--TGGATACCC--CAATTAGTA------
(Ch)Ixodes uriae                   -ACTAATTTAGCAAAACTATAA-TGTATC-AATTTTAGAATTTGAAGA--TGGTAATAC--CAATTAGTA------
(Ch)Leptotrombidium deliense       ---TAGTGATGAAACATTTTAGATGTAATAGGACTTAAGATCTTATAAACTATTTTTTA-AGCTCACTTA------
(Cr)Geothelphusa dehaani           -GTTATTTTAGCAAGGTT-----TGCATT-AAACTTAAGATTTAAATACATAGATTCGT--CTATAAGTAATA---
(Cr)Pollicipes mitella             -ACTGATGTGGCAGACA---AG-TGCTAT-AGATTTAAGATCTATATATAAAGGTTATAATCCTTTTTTCAGGA--
(Fi)Aphyocypris chinensis          -GCTTCCGAAGGATAACAGTTCATCCATT-GGTCTTAGGAACCAAAAACTCTTGGTGCA-AATCCAAGCAGAAGCT
(Fi)Carassius auratus x Cyprinu    -GCTTTTAAAGGATAACAGTTCATCCGTT-GGTCTTAGGAACCAAAAACTCTTGGTGCA-AATCCAAGTAGAAGCT
(Fi)Gonorynchus greyi              -GCTCCTAAAGGATAACAGTTCATCCATT-GGTCTTAGGAACCAAAAACTCTTGGTGCA-AATCCAAGTAGTAGCT
(He)Anabrus simplex                -ACTACTTTGGCAGAGAAG----TGTATT-GAATTTAGAATTCAAAAATGTAAGAGTAT--T-ACAGGTAGTA---
(He)Bactrocera dorsalis            -ACTATTTTGGCAGATT---AG-TGCAAT-AAATTTAGAATTTATTTATGTAATTTATA--TTACAAATAGTA---
(He)Homalodisca coagulata          -GCTAGATTGGCAGATTTT-AG-TGCAAT-AAATTTAGAATTTATAAAAATGATTAAT---TCATATCTAGTA---
(Ma)Chlorocebus sabaeus            -ACTTTTAAAGGATAACAGCTA-TCCATT-GGTTTTAGGAACCAAAAATA-TTGGTGCA-ACTCCAAATAAAAGTA
(Ma)Choloepus didactylus           -ACTTTCATAGGATAAAAGCAA-TCCATT-GGTCTTAGGAACCAAAAA-A-CTGGTGCA-ACTCCAGATGAAAGTA
(Ma)Echymipera rufescens austra    -ACTTTTAAAGGATAAAAGTAA-TCCATT-GGTCTTAGGAACCAAAAATT-TTGGTGCA-ACTCCAAATAAAAGTA
(Mo)Acanthocardia tuberculata      ---TCCCACGTAGTAGAATATAATATATT-GGATTTAGGTTCCAAAGTTGAGCAGTGC----TCCATGGGAT----
(Mo)Dosidicus gigas                -ACAGAGTTGGCAGAAAT---A-TGTGAA-TAATTTAGGTTTATTTTATGAGATTTATAAATCTCACTTTGTA---
(Mo)Pupa strigosa                  AGATAG-GTGGCAGAGTAG-CA-TGCACT-GGCTTTAAGCGC-CAAATACGGGATTTTATAGGCCCCTTATCTT--
(Re)Cuora aurocapitata             -ACTTTTAAAGGATAGAAGCAA-TCCACT-GGTTTTAGGAACCATTAACCCTTGGTGCA-ATTCCAAGTAAAAGTA
(Re)Geochelone pardalis            -ACTTTTAAAGGATAGAAGTTAATCCGCT-GGCTTTAGGAGCCAGTCACCCTTGGTGCA-ACTCCAAGTAAAAGTA
(Re)Pelomedusa subrufa             -ACTTTCAAAGGATAGAAGTTA-TCCAAT-GGCTTTAGGGGCCATCTTCCCTTGGTGCA-ACTCCAAGTGAGAGTA
```

```
tRNA-Lys
(Am)Aneides hardii               CATTA-AGAAGCTTTCACGGATA--AGCAACAGCCTTTTAAGCTGAAAC-----TCGGTGGCCTCCA-A-TCACCC
(Am)Bombina variegata            CACTA-AGAAGCTAATACGGAAT--AGCAACAGCCTTTTAAGCTGAAGA-----CAGGTGGC-CCTA-A-TCACCC
(Am)Microhyla ornata             CATTA-AGAAGCT---ATAGGAC--AGCGACAGCCTTTTAAGCTGTAGA-----TAGGTGAT-TCCA-A-CCACCC
(Bi)Ciconia boyciana             CATTA-AGAAGCT---ATGCAAA-CAGCACTAGCCTTTTAAGCTAGAGA-----TAGAGGACTACCC-ACTCCTCC
(Bi)Phaethon rubricauda          CATTA-AGAAGCT---ATGTAT--CAGCACTAGCCTTTTAAGCTAGAGA-----AAGAGGCCCGCTA-G-TCCTCC
(Bi)Pteroglossus azara flaviros  CATTA-AGAAGCT---ATGTAC--CAGCACTAGCCTTTTAAGCTAGAGA-----AAGAGGGCCACC----CCCTCC
(Ch)Habronattus oregonensis      AGATA-GATGGCCGAATTAT----AGGTGTTAGTCTCTTAAATTAATT-------ATG-----------------
(Ch)Heptathela hangzhouensis     CTTCA-GATAACCTAAATA-----AGGTATTGGCCTCTTAAGCCTAATT-----ATAGAATT---------TCTT
(Ch)Mesobuthus martensii         GACTA-GGTGGCTGAGGGAA----AAGCGTTGGTCTCTTAAATCAATT------TACGGTAT-----AA---ACCT
(Cr)Macrobrachium rosenbergii    CATTA-GGTGACTGAAAGC-----AAGTGTAAGTCTTTTAAACTTACA------ATAGTAGTT--ACGC-CTACTT
(Cr)Pseudocarcinus gigas         CACCC-GATGACTGAAA-GT----AAGTGTAGGTCTTTTAAACCTATC------ATAGTAAC-----CGCCTACTT
(Cr)Speleonectes tulumensis      AATTA-GATGGCTGAAAAACCA--AAGCAATGGTCTCTTAAACCATTATTAACAATAGTAGACCAAGGTACTACTT
(Fi)Acanthogobius hasta          CGCTATAGAAGCTAAACAGGACGATAGCGTCAGCCTTTTAAGCTGAAGA-----ATGGTGCCCTCCA-A-ACACCC
(Fi)Glossanodon semifasciatus    CATTA-AGAAGCTAAATCGGGAA-TAGCGTTAGCCTTTTAAGCTAAAGA-----CTGGTGGCCCCCA-A-CCACCC
(Fi)Sardinella maderensis        CACTA-GGAAGCTAAATCGGGCC-TAGCGTCAGCCTTTTAAGCTGAAGA-----TTGGTGACCCCCA-A-CCACCC
(He)Bactrocera carambolae        CATTA-GATGACTGAAAGC-----AAGTACTGGTCTCTTAAACCATCTT-----ATAGTAAATTAGCAC-TTACTT
(He)Bemisia tabaci               CATTA-AAAAGCTTAAAGT-----AAGTGTTGATCTTTTAAATCAATT------ATGGTAAT---GTCGTCTATCT
(He)Melipona bicolor             CATTA-GATGTCTGATAAA-----AAGAATTAATTTTTTAAATTAAAAT-----ATAATAAT---GAATATTATTT
(Ma)Cavia porcellus              CATTA-CGAAGCT------AATTGCAGCGTTAACCTTTTAAGTTAAAGA-----CCGGGGCC--CAA-A-TCCCCC
(Ma)Pan paniscus                 CACTG-TAAAGCTAACC-------TAGCATTAACCTTTTAAGTTAAAGA-----TTAAGAGGACCAACACCTCTTT
(Ma)Tarsius bancanus             CACTG-CGAAGCTTATA-------TAGCATTAACCTTTTAAGTTAAAGA-----CCGAAAGTACTAA--TCTTTCC
(Mo)Acanthocardia tuberculata    -GTCGTGGTAGCTTAATTTCTAA--AGCGCGGGCCTTTTAAGCC--TGA-----TATAATTGTATTG----ATTCC
(Mo)Lampsilis ornata             TCCAC-GATGGCCGAGA-AC----AGGCATCGAGCTTTTAACTCGACT------ACAGTCAC-----C----ACTT
(Mo)Mizuhopecten yessoensis      -GGGTTGGTAGCCTAATGC--AG--GGCGGCGGACTTTTAATTCGTAGG-----TGTAGTGTAAAAA----GCTTC
(Re)Gekko vittatus               -TTTAATGTAGCTAAACACTATA--AGCACTGGCCTTTTAAGCCAGTAA-----TGGG-----CCCATTGCGCCCC
(Re)Sceloporus occidentalis      CACTA-AGAAGCT---TTATA---CAGCACTAGCCTTTTAAGCTAGAGA-----AGGGGAATCACC----TCCCCC
(Re)Testudo kleinmanni           CACTA-AGAAGCTAAACAGGG---TAGCGCTAGCCTTTTAAGCTAGAAA-------AAGAGAACCTCCAACTCTCC
```

```
tRNA-Met
(Am)Ambystoma californiense      AAGTAAAATAAGCTAAAT---AAGCTTTTGGGCCCATACCCCAAATATGTTGGTTAAACCCCTTCTTTTACTA
(Am)Xenopus laevis               -AGTAAAGTCAGCTAAAA---AAGCTTTTGGGCCCATACCCCAAACATGTTGGTTAAACCCCTTCCTTTACTA
(Am)Xenopus tropicalis           -AGTAAAGTCAGCTAAAA---AAGCTTTTGGGCCCATACCCCAAACATGTTGGTTAAACTCCTTCCTTTACTA
(Bi)Cygnus columbianus           -AGTAGGGTCAGCTAATCA---AGCTATCGGGCCCATACCCCGAAAATGATGGTTCAACCCCCTCCCCTACTA
(Bi)Dryocopus pileatus           -AGCAAGGTCAGCTAAAAA---AGCTATCGGGCCCATACCCCGAAAATGAAGGTTCAACCCCCTCCCTTGCTA
(Bi)Falco sparverius             -AGTAGAGTCAGCTAAACACAAAGCTATCGGGCCCATACCCCGAAAATGATGGTTTAACCCCTTCCTCTACTA
(Ch)Heptathela hangzhouensis     -ATAAAAGTAAGCTAATT--AAAGCTTGTGGGTTCATACCCC----ATTTATGGTT-TTCCCTTTTATA----
(Ch)Leptotrombidium akamushi     -AGAAAGGTAAGCTATAT--CAAGCTAGCGGATTCATAATCCTTTGAAACATTTTTATGTCCTTTCCT-----
(Ch)Mesobuthus gibbosus          -TTAAAGGTAAGCTAATTTTGAAGCTGTTGGGTTCATACCTC----GAAAATGAATATTCCCTTTAAT-----
(Cr)Geothelphusa dehaani         -TGAAAGATAAGCTAATC---AAGCTAATAGGTTCATACCCTGTAAATGAAAGTT-CACACTTTCTCTTTCTA
(Cr)Litopenaeus vannamei         -AAAAAGATAAGCTAAGC---AAGCTCGTGGGCTCATACCCCATCTATGAGGGTCATACTCCCTCTCTTTTTA
(Cr)Panulirus japonicus          -AAAAAGATAAGCTAATTT-AAAGCTAACGGGCTCATACCCCGTCTATGTGTGTC-TACTCACACTCTTTTTA
(Fi)Diagramma pictum             -AGTAAAGTCAGCTAACGC--AAGCTTTCGGGCCCATACCCCGAACATGTAGGTTCGATTCCTTCCTTTGCTA
(Fi)Hypentelium nigricans        -GACAAAGTCAGCTAAT-T--AAGCTTTCGGGCCCATACCCCGAACATGACGGTTAAAATCCCTCCTTTGTCA
(Fi)Ostichthys japonicus         -AGTAAAGTCAGCTAAATT--AAGCTTTTGGGCCCATACCCCAAACATGTTGGTTAAATTCCTTCCTTTACTA
(He)Aleurochiton aceris          -AGATTAATAAGCTAAG-ATAA-GCTATTGGATTCATACTCCAATTATAAATTT----ATATTTTTATTCTA-
(He)Neomaskellia andropogonis    -AGAATAGTAAGCTAAATATAA-GCTATTGGACTCATATTTCAAGTATAGCATA----AG--TTTTATTCTA-
(Ma)Lemur catta                  -AGTAAGGTCAGCTAAACA---AGCTATCGGGCCCATACCCCGAAAATGTTGGTTTATATCCTTCCCGTACTA
(Ma)Leptonychotes weddellii      -AGTAAGGTCAGCTAAATA---AGCTATCGGGCCCATACCCCGAAAATGTTGGTTTATCCCCTTCCCGTACTA
(Ma)Phoca largha                 -AGTAAGGTCAGCTAAATA---AGCTATCGGGCCCATACCCCGAAAATGTTGGTTTATCCCCTTCCCGTACTA
(Mo)Acanthocardia tuberculata    -CGGGGGATAGGATAAGTTAAAATCCGGCGGGTTCATACCCCGCATATACCGCTGGTTCCCCCGT--------
(Mo)Ilyanassa obsoleta           -TGTAAAATAAGCTAAATATAAAGCTATTGGGTTCATACCCCAAAAATGAACAC----GAGTTCTTTTACAA-
(Mo)Vampyroteuthis infernalis    -AATAAAGTAAGCTAAATGTAA-GCTATTGGGTTCATACCCCAAAAATAGATTTTTAAAAATCTTTTTATTA-
(Re)Boa constrictor              -AGTAAAGTCAGCTAAATA---AGCTATCGGGCCCATACCCCGAAAATGCCACTACGGCCTTCACTA------
(Re)Chrysemys picta              -AGTAAAGTCAGCTAACT---AAGCTATTGGGCCCATACCCCAAAAATGTTGGTTTAAACCCCTCCTTTACTA
(Re)Manouria emys                -AGTAAAGTCAGCTAAT-T--AAGCTTTCGGGCCCATACCCCGAAAATGTTGGTTAAAATCCTTCCTGTACTA
```

```
tRNA-Phe
(Am)Ambystoma dumerilii            GTTAATGTAGCTTAAA---TAAAAGTGTGACACTGAAAATGCCAAGATAGAT--CTTAAAACATCTCATAAACA-
(Am)Buergeria buergeri             ACCCTTATAGCTTAAC----CAAAGCGTAGCGCTGAAAACGCTAAGACGGAC--CCTAAAAAGTCCTGAGAGTA-
(Am)Rhyacotriton variegatus        GTTCACGTCACTTAAT---TTAAAGTTTGATACTGAAGACATCAAAATAGGT--CTAAATAGGCCTCGTAAACA-
(Bi)Ardea novaehollandiae          GTCCTTGTAGCTTAAT--ACCAAAGCATAGCACTGAAGATGCTAAGATGGATGCC---CACATTCCCAAGGACA-
(Bi)Melopsittacus undulatus        GTCCCTGTAGCTTAA---ACTAAAGCATGGTGCTGAAGATACCAAGATGGACCCC---CCC-CTCCCAAGGACA-
(Bi)Vidua chalybeata               GTCTTTGTAGCTTATA--A--AAAGCATGACACTGAAGATGTCAAGATGGTCGTTACACACACACCCAAAGACA-
(Ch)Amblyomma triguttatum          ATTTTCATAGTTTAAG--A-AAAAACATTACACTGAAAATGTAAAAATAAT-----TATT-----TTGAAAATA-
(Ch)Ixodes persulcatus             ATTCTTATAGTTTAAT--ATAAAAATATTACACTGAAAATGTAAAGAAATA-----TTTT-----TTAAGAATA-
(Ch)Leptotrombidium pallidum       TTTTGTATAGCTTATA----TAAAGTATTGTTTTGAAGAAACAAAGAAGAAACAATTC-----------------
(Cr)Hutchinsoniella macracantha    GCTCATATCAAT--------AAGAGCATAACATTGAAGCTGTTAAGA--AA-ATCC----AAGATTCATA-AATA
(Cr)Speleonectes tulumensis        TTTCATATAGTCTAAT--G-TAGAATTTAACCCTGAAGAGGTTAAGGTAGTG----TATCGGTGCTTGTGAATA-
(Cr)Vargula hilgendorfii           ACTTATATAGCTTAAG---TAAGAGTGTGGTTTTGAAGCGGCCGAGGTAGTAG-------TATACTTATGAGTA-
(Fi)Anguilla dieffenbachii         GTTAACGTAGCTTAAA--CAAAAAGCACGGCACTGAAGATGCCAAGATGAGC--CATAAAAAGCTCCGATGACA-
(Fi)Chlorophthalmus agassizi       GCTTACGTAGTTTTAC--TAAAAA-TTTAACACTGAAGATGTTAAGACGGAC--CCTAAATAGTCCCGTGGGCA-
(Fi)Gasterosteus aculeatus         GCTAGCGTAGCTTAAC--TAAAG--CATAACACTGAAGATGTTAAGATGGGC--CCTAGAAAGCCTCGCAAGCA-
(He)Bactrocera carambolae          ATTCAAGTAGCTTAAA---ATAGAGCATAACACTGAAGATGTTAGGGTAATTGAA------TAATTCTTGGATG-
(He)Bothriometopus macrocnemis     -CTTCTGTAAGCTC-----TCAGAGCGAAACATTGAAGATGTTTAGGCGAAGAATC----CATCTTCACGGAAGA
(He)Campanulotes bidentatus com    GCTTATATAATTT-------GAAAGTGTGACTTTGAAGGTGTTAAGA--AGGATGT----AGGGTTCATCTAGCA
(Ma)Macroscelides proboscideus     GTTAATGTAGCTTAAC-TGACAAAGCAAGGCACTGAAAATGCCTAGATGAGTGATTTCCCCAACTCCATAAACA-
(Ma)Phalanger interpositus         GTTAATGTAGCTTAAT---ACAAAGCAAAGCACTGAAAATGCTTAGATGGACT-TTAACATAGTCCCATAAAC--
(Mo)Ilyanassa obsoleta             GCCTTGATAGCTTAACTTTTTAGAGCATAGCACTGAAGATGCTAGGGTGGCAATTA----TTGTCTCAAAGCA--
(Mo)Nautilus macromphalus          ACTTATTTAGCTTA-----TAAGAGCGCAGCGTTGAAGGTGCTGAGGAGGTTGATG----TAAACTAATATGTA-
(Mo)Vampyroteuthis infernalis      ATCTAAGTAGCTTAAA----TAGAGCGTAACGTTGAAGGTGTTAAGGTAATTAAAAA-TAATAATTTATAGATA-
(Re)Iguana iguana                  GTTATTGTAGCTTAATTTATCAAAGCACGGCACTGAAAATGCCGCGATGGGCTACAAAAAAAGCCCCAAAAACA-
(Re)Macroclemys temminckii         GTTATTGTAGCTTA---TTACAAAGCATGGCACTGAAGTTGCCAAGATGGATTGTCACCAT-ATCCCAAAAACA-
(Re)Mauremys mutica                -TTATTGTAGCTTA---TCGTAAAGCATGGCACTGAAGTTGCCAAGATGGGTAATCAACAT-GCCCCAAAAACA-

tRNA-Pro
(Am)Ambystoma laterale             CAAGAGGTAGTTTGATTCCAAGAATTTTGGCTTTGGATGCCAGTGGAGGAGGTGAAAATCCTCCCCTCTTGA-
(Am)Andrias japonicus              CAAGAGATAGTTTAGGTTCCAGAATTCTGGCTTTGGAGGCCAGGGGTAAGGGTGAAAATCCCTTTTTCTTGA-
(Am)Aneides hardii                 CGGGCGATAGTTTAGTTTTAA-AACCTTGGCTTTGGGTGCCGAAAAAGGGAGGGCGGAACTCCTTTACCCGA-
(Bi)Arenaria interpres             CAGAAAATAGTTTAG-TGTAAAA-TACCAGCTTTGGGAGCTGGAGACAGAGGTTTGAGCCCTCTTTTTCTGA-
(Bi)Cathartes aura                 CAGAGAATAGTTTAG-TGTAAAA-TACCAGCTTTGGGAGCTGGGGATAGAGGTTTAAGTCCTCTTTTTCTGA-
(Bi)Smithornis sharpei             CAGAAAGTAGTTTAG-TTTAAAAATGCCAGCTTTGGGAGTTGGAGATAAAGGTGTGATTCCTTTCTTTTTGA-
(Ch)Leptotrombidium pallidum       CAGAAAATAGTTTAT-T--GAA--TATTCACTTTGGGGGTGAAAGG-------------ATT--TATTCTGA-
(Ch)Mesobuthus gibbosus            CAGAGAATAGTTAAA-T--AGACAT-TTAGATTTGGAGTCTAAAGATA---------TTTCTATTTTTCTGA-
(Ch)Metaseiulus occidentalis       CAAAAAATAAGTTAA-ATAAAAACTATAAATTTTGGAGATTTAAATTGAAA--------ACTTCTTTTTTGA-
(Cr)Armillifer armillatus          CAAAGGATAGTTTAT-TTAGAA--TATTAATTTTGGGGATTGAAGG-------------ATTGTTCTTTTGA-
(Cr)Harpiosquilla harpax           CAAGGGGTAGTTTAA---ATAAAATGTTAGTTTTGGGAATTAACGATAGGAAGT--GTT-TCCTTTCCTTGA-
(Cr)Panulirus japonicus            CAGGAGGTGGTTTAA---GTAAAATATTAATTTTGGGGATTAAAGATAGGGTAT--GTTGCCTTTTTCCTGA-
(Fi)Ijimaia dofleini               CAGAGAATAGTTTAA-TTAAGAATCT-TAGCTTTGGGAGTTAAGGGTGGGAGTTAAAATCTCTTTTCTTTGA-
(Fi)Oncorhynchus mykiss            CAGAGGGTAGTTTAA-TTTAGAATCT-TAGCTTTGGGAGTTAAGGGTGGGAGTTAAAATCTCCTCTCTCTGA-
(Fi)Sardina pilchardus             CAAAGAATAGTTCAA-CTTAGAATATCTAGCTTTGGGAGTTAGGGGTGGAAGTTAGAATCTTTCTTCTCTGA-
(He)Aleurodicus dugesii            CAGTAGATAATTTAA-AAAAAAAATATTAATTTTGGAAATTAAAAATAAAA-----TTATTTTTCTACTGA-
(He)Homalodisca coagulata          CAGCAAATAGTTTAT-TAAAA---TATTAATTTTGGAGATTAAAGATGTAA------ATTCTACTTTGCTGA-
(Ma)Isoodon macrourus              CAGGAAGTAGTTTAA---GTAGAATATCAGCTTTGGGTGTTGATGGTGAGGTGGTTAG--CCTTCTTCTTGA-
(Ma)Rattus norvegicus              CAAGAAGTAGTTTAA---ATAGAATATCAGCTTTGGGTGTTGATGGTGGGGAGGTAGTT-CCTTCTTCTTGA-
(Ma)Semnopithecus entellus         CAGAAAGTAGTTTAG---ATAGAACGTCAATTTTGGGTATTGGTGGCGAAGCTA--GGTGCTTTCTTTCTGA-
(Mo)Cepaea nemoralis               TTAAAAGCAGTATTA---G-AATATGGCAACTTTGGAAGTTGGTGATTAGA------GCGATCTACTTTTAAA
(Mo)Nautilus macromphalus          CAGAAGGTAGTTTAT---GTAAAATTTTAATATTGGAAATTAGTGATTGAGGAA--GGC-CTTATCTTCTGA-
(Re)Gekko gecko                    CAAAAAATAGTTTAA-TTAAAACAC-TT-GTTTTGGGGACAAGTAATGGGCACT---TTTGTGGCTTTTTGA-
(Re)Lepidophyma flavimaculatum     CAAAAGGTAGTTTA----TAAAAATGCTAGTTTTGGGGGCTAGCGATGGGCTGTTTGATGGCCTCCTTTTGG-
(Re)Varanus niloticus              CAGAAGGTAGTTTAA-GTGTAGAATTCTAGTTTTGGGGACTAGGGGTGAGAGTT------TCTTTCTTCTGA-
```

```
tRNA-Ser
(Ma)Acinonyx jubatus            -TTGAGAAAGACA-TAATGGTT--ATGGCATTGGCTTGAAACCAGTTAAAGAGGGTTCGATTCCTTCCTTTCTT--
(Ma)Cavia porcellus             ---AAGAGGGTCA-TAAAGGTT--ATGGGGTTGGCTTGAAACCAGCTTTAGGGGGTTCAATTCCTTCCTCTCTTG-
(Am)Ambystoma mexicanum         -AAGAAAGTGACA-GATTGGTT---TGTGGTTAACTTGAAATTAATTTAAGGGGGTTCAATTCCCTCCTTTCTTG-
(Am)Batrachoseps wrightorum     -AAGGAAGTGGCA-GATTGGCT--ATGTATTTAACTTGAAATTAAAAAACGGGGGTTCAATTCCTTCCTTCCTT--
(Fi)Anguilla celebesensis       -AAGAAAGTGGCA-GAGTGGTT--ATGCGGCTGGCTTGAAACCGGCAAATGGGGGTTCAATTCCTTCCTTTCTTG-
(Bi)Ardea novaehollandiae       -AAGAAAGAAGCATGAGTGGTTTAATGCGGTTGGCTTGAAACCAGCGTATGAGGGTTCGATTCCTTCCTTT-----
(Re)Lepidophyma flavimaculatum  -GAGAAATGAGTATAGGTGGCT--GTACGGTTGGCTTGAAACCAGTT-ATGGTGGTTCGACTCCACCTTTTCTT--
(Re)Bipes biporus               --GAGAAGGAGCA-TGGCGGTC--ATGTGCCTGGCTTGAAACCAGTGTTTGGTGGTTCAATTCCATCCTTCTTCG-
(Bi)Crocodylus niloticus        -AAGGACAGAGCA-TGTCGTAT---TGCGGTTGGCTTGAAACCAAATGGTGGGGGTTCAATTCCCCCTGTCC----
(Ch)Ornithodoros moubata 2      -------GACATTTTAACTATTTAAGTAT-ATATTTGAAAATATAA-AAAAGAATT--CATC--TAAATGTCT--
(He)Artogeia melete 2           ------AATTAATGAGCTTGTAAAAGCAT-TTGTTTGAAAACTTAA-GAAAGAATTATTATT--CTATTAATTT-
(Cr)Daphnia pulex 2             ------GATTGTTTAACCTAATTAAGGTTCTTGTTTGAAAGCAAGTCATAAAAGTTAAAATCTTTTAGCACTCT-
(Mo)Haliotis rubra 2            ------AGCTGCT-GGCCTGGGGCAGGTT-TTGTCTTGAAAATGAAACAGAAGTGTTCGAATCACTTAGTAGCTT-

(Ma)Acinonyx jubatus 2          ----------GAAAAAGTATGCAAGAACTGCTAATTCATGCCT-CCACGTATAAAAA-CGTGGCTTTTTCA-----
(Ma)Cavia porcellus 2           -----------AGGAAGACAACTGGAACTGCTAATTCCTCCCT-CCATATTTAAAAA-TATGGCTTCCTT------
(Bi)Crocodylus niloticus 2      -------CATGAGAGTGCCTACAAGAACTGCTAATTCTGTCCC-CCGGATTTAATCA-CCCGGCTCTCATATTACA
(Re)Lepidophyma flavimaculatum  ---GAGGGGCGTACAACACAAAAAGACCTGCTAAGTCTTCCTA-CTGTGGTTAACCC-CCCAGGCCCCTT------
(Bi)Ardea novaehollandiae 2     --GAGGGGAGGTTCAAC-CAACAAGAACTGCTAACTCTTGTAT-CTGAGTCTAAAAC-CTCAGCCCCCTTA-----
(Re)Bipes biporus 2             ---AAGAGGCGTATGAA-CAACGAGAGCTGCTACCTCAAGAAA-CCGAGACTAAACTTCCCGGGCCTCTT------
(Am)Ambystoma mexicanum 2       -----------AAAGAAACAAGAGAAACTGCTAATATCTTAAT-CTGTAGTTAAAATCCACAGTCTACTCA-----
(Fi)Anguilla celebesensis 2     GAGAGAGTAAGACACAAGCCTGAAGAATTGCTAGTCCTTCAAGACCGTGGTTTAAATCCACGGCTCACTCG-----
(Am)Batrachoseps wrightorum 2   --GAGAAGAATTAAGAAATGT-AGAAACTGCTAATTATCTAAC-CCGTGGTTAAACTCCACGATCTACTC------
(Ch)Ornithodoros moubata        -----------AAAGCTAAAAACCAGGCTGCTAACCTGATAATAATGATACTCA----TTTAGCTTTT--------
(Cr)Daphnia pulex               ------GAAGTAAAAGTAAGGGAAGAGCTGCTAACTCTGTCTCTAAGCGGTTGAACTCCGTTTGTATTTCT-----
(He)Artogeia melete             ---------GAAATATTTTATATTAAGCTGCTAACTTAATTTT--AGTGGTTAATTACCATTAATATTTCT-----
(Mo)Haliotis rubra              -CGGGGGAAACCAGGGGTAGAACAGGGCTGCTAACTTTGTTTT-GGGTGGTTCGGC-CCATCCTTCCCCCTA----

tRNA-Thr
(Am)Bufo melanostictus          GCCTCAGTAGCTTAATAC--TAAAAGCATTGGTCTTGTAAACCAAAGAATGAAGAATAAACCCTTCCTGAAGCT--
(Am)Hemidactylium scutatum      ACTGACGTAGTTTAAA------AAAACACTGATTTTGTAAATCAGAAAATGG----AGCTTAAAACTCCCGTCCGT
(Am)Hynobius formosanus         -CTTAGATAGCTTAAA-----TAAAGCGTCGGTCTTGTAAACCGAAAATGGGGAATAACCCCCCCTCCAAGT----
(Bi)Pandion haliaetus           ACTCTAATAGTTTA-T-----AAAAACATTGGTCTTGTAAACCAAAGAATGAAGACTGCCCCTCTTCTTAGAGTT-
(Bi)Smithornis sharpei          ACTCTAATAGTTTA-G-----AAAAACATTGGTCTTGTAAGCCAAAGATCGAAGACT-TACCCCTTCTTAGAGTT-
(Ch)Heptathela hangzhouensis    GCTCTAATAGTTTAATT-----AAAATATTGATTTTGTAACTCA-AAGATCC----ATTTTGGAGCTTCAG-----
(Ch)Nothopuga sp. 1 LP-2008     GTTTTGATAGTTTAAA----ATAAAACCATGGTCTTGTAAACCATAAATGAA--------TCCATTCTCAAAACT-
(Ch)Ornithodoros porcinus       ACTATAATAGTTTAA-----AAAAAACAAAGGTTTTGTAAACCTTAATTG-----------AAATTCTTATAGTT-
(Cr)Argulus americanus          GCTTTAGTAGTTAAAT-------TAACGTTAATTTTGTAAATTA-AAAATGGTCTTACCCTAAAGCT---------
(Cr)Tetraclita japonica         GTCTATGTAATTTATT-----AAAAATACTGGTCTTGTAAATCAGAAA--AAGGATTTTACC---TCTTAGGCT--
(Cr)Triops longicaudatus        ACCTTAATAGTTTAA-----AAAAAACCCTGGTCTTGTAAATCAGAAATAAGA------TTAGTCTTTTAAGGT--
(Fi)Kentrocapros aculeatus      GCATTAGTAGCTCAGAC---TCAGAGCGCCGGTCTTGTAAACCGGATGTCGAGGGTTAGAATCCCTCCTACTGCT-
(Fi)Neoscopelus microchir       GCCCTAGTAGCTCAGTGAC-TTAGAGCATCGGTCTTGTAAGCCGAACGCCGGAGGTTAAAGTCCCCCCTAGTGCT-
(Fi)Scopelogadus mizolepis      GCCCTAGTAGCTCAGTG---TTAGAGCACCGGTCTTGTAAACCGGACGCCGGAGGCTAAATTCCTCCCTAGCGCT-
(He)Oxya chinensis              GTTTTAATAGTTTAAT----A-AAAACGCTGGTCTTGTAAACCAGAAATAAGAATAGACCCCCACTTTTAAAACT-
(He)Reticulitermes flavipes     GTTTTAATAATTTAAT----A-AAAATATTGGTCTTGTAAACCAAAAATAAGGAA-----TCAACTTTTAAAACT-
(He)Trialeurodes vaporariorum   ATTTTAATAGTTTAAT----AAAAAACTTTGATTTTGTAATTCAAAAATAC---------TCATCGTTTAAAATC-
(Ma)Myoxus glis                 GTCCTGGTAGTATAAAG---TTATTACTCTGGTCTTGTAAACCAGAAATGGAAATC----TCAATTTTCCCCAGGA
(Ma)Oryctolagus cuniculus       GTCCCAGTAGTATAAA-----TATTACTCTGGTCTTGTAAGCCAGGAATGGAGAT-------AGTTCTCCCTGAGA
(Ma)Petaurus breviceps          -------TAATTTAAC-----CAAAATACTGGCCTTGTAAGCCAGCAACGAAGGAAAATACCC-TTCCTAGGACA-
(Mo)Conus textile               GCAACGATAGCTTAAA----TGAAAGTATTGGTCTTGTAAGCCAA----TGATGAACTAAACGTTCTCGCTGCT--
(Mo)Nautilus macromphalus       GCCCTGGAAGCTTGT-----TTTAAGTAGCGGCCTTGTAAACCGAAGATTGTGATACTAAATCTCTCAGGGCA---
(Mo)Watasenia scintillans       GCTATAATAGTTTAAA-----TAAAACTTTGGTCTTGTAAACCAAAATTAGATCTC----TATATCTTTATAGTT-
(Re)Chlamydosaurus kingii       GCTCTGCTAGCTTAAACACCACAAAGCACTAGTTTTGTAAACTAAAGATGGGGAC------ACCCGCAGGTCA---
(Re)Iguana iguana               GCCCTAGTAGCTTAACCTC--TAAAGCATTGGTCTTGTAAACCAAAGCTGGGACTTAAACCGCCCCTAAGGCA---
(Re)Shinisaurus crocodilurus    GTCTTAATAGCTTAACCTC-ATAAAGCACTGGTCTTGTAAACCAGAGCCGGGTAT--ACTTACCCTTAAGACA---
```

```
tRNA-Trp
(Am)Ambystoma californiense       --GGATTTAAGATAA---TC---AGACTAAAGACCTTCAAAGCCTTAAGTAGAAGTTT---AAACCTTCTAATCCT
(Am)Ambystoma mexicanum           -AGGATTTAAGATAA---TC---AGACTAAAGACCTTCAAAGCCTTAAGTAGAAGTTT---AAACCTTCTAATCCT
(Am)Xenopus laevis                AGAGATTTAAGTTAACA------AGACTAAGAGCCTTCAAAGCCCTAAGCAGGAGTTA---GAATCTCCTAATCTC
(Bi)Micrastur gilvicollis         AGAAACTTAGGATAGCCCCT---AAACCGAAGGCCTTCAAAGCCTTAAACAAGAGTTA---AACTCTCTTAGTTTC
(Bi)Pandion haliaetus             AGAAACTTAGGATAACCA-----AAACCGAAGGCCTTCAAAGCCTTAAACAAGAGTTA---AACCCTCTTAGTTTC
(Bi)Podiceps cristatus            AGAAACTTAGGATAACCC-----AAACCGAAGGCCTTCAAAGCCTTAAACAAGAGTTA---AACCCTCTTAGTTTC
(Ch)Habronattus oregonensis       AGGATATTAAGATAATAATT------CTATTAGCCTTCAAAGTTAA--AAGTACT---------------------
(Ch)Leptotrombidium akamushi      AGGACTCTAGGTTAA--ATT---AAACTTTTGGCTTTCAAATCCAAAGATCGGCAGTT---CAA------------
(Ch)Mesobuthus gibbosus           TAAGTGTTAAGTTAA--GA----AAACTAGAGGCCTTCAAAGCCTC--AATTGGTGAG---AGCTATACTTTA---
(Cr)Armillifer armillatus         AGGACTTTAAGTTAA--AAT---AAACTATCAACCTTCAAAGCTGG--AAATCTATAA---GTCTTA---------
(Cr)Callinectes sapidus           AGGATTTTAAGTTAT---TT---AAACTAAAGGCCTTCAAAGCCTAAAAAAAAAGA-T---CATCTTTTAAATCCT
(Cr)Portunus trituberculatus      AGGATTTTAAGTTAT---AT---AAACTAAAGGCCTTCAAAGTCTAAAAAAAAAGAAT---TCTCTTTTAAATCCT
(Fi)Engraulis encrasicolus        -GAGACTTAGGATAGCATTT---AGACCATGAGCCTTCAAAGCTCCGAGCAGGAGTGA---AAATCTCCTAGTCCC
(Fi)Katsuwonus pelamis            AGGGACTTAGGATAGTAAAT---AGTCCAAGGGCCTTCAAAGTCCTAAGCGAGGGTGA---AAATCCCCCAGCCCC
(Fi)Triacanthus biaculeatus       AGAGACTTAGGCTAGCAACCC--AGACCAATGGCCTTCAAAGCCATCAGCGGGGGTGA---AAATCCCTCAGTCCC
(He)Bactrocera oleae              AAGGCTTTAAGTTAA--TC----AAACTAATAGCCTTCAAAGCTATAAATATAAGTTT---AATCTTTTAAGCCTT
(He)Gomphiocephalus hodgsoni      AAGACTTTAAGTTAA--TA----AGACTATTAGCCTTCAAAGCTTTAAATATAAGTTT---AATCTTTTAAGTTTT
(He)Lepidopsocid sp. RS-2001      AAAATCTTAAGTTAA--TTT---AAACTAATAACCTTCAAAGTTAT--ATATAAATTT---ATATATTTAGATTTT
(Ma)Callorhinus ursinus           AGGGATTTAGGCTAAACC-----AGACCAAGGGCCTTCAAAGCCCTAAGTAAGTT-TC----ACTAACTTAATCCC
(Ma)Chrysochloris asiatica        AGGGGTTTAGGTTCATTC-----AGACCAAAAGCCTTCAAAGCTTTAAGAAAGTGATA------TCACTTAACCCC
(Ma)Episoriculus fumidus          AGGAATTTAGGTTAGATC-----AGACCAAGAGCCTTCAAAGCTCTAAGCAAGTAGTT----ACTCACTTAATTCC
(Mo)Conus textile                 AAGGTCTTAAGTTAT---TA---AAACTGCAGGCCTTCAAAGCCTGAAATAAAGA------AGATTCTTTAGTCCT
(Mo)Crassostrea gigas             TGGACCATAAGTTAAGGATT---AAACTGTCAAACTTCAAATTTGA--AACTACTCAT---AATTGGGTTGGTCTA
(Mo)Octopus vulgaris              AGAGTTTTAAGTTAA--ATT---AAACTAAAAGTTTTCAAGACTTT--AAATAG--AT---AATTATATTTAAATT
(Re)Abronia graminea              AGAAATTTAGGTTACATC-----AAACCAAGGGCCTTCAAAGCCCTAAACAAGGA------AAGCTCCTTAATTTC
(Re)Bipes canaliculatus           AGAAGCTTAGGTTACGCCATTTTAAACCAGAGGCCTTCAAAGCCCCAAACAAGAC-----CAAACGTCTTAGCTTC
(Re)Testudo kleinmanni            AGAAACTTAGGATTAATCCTACAAAACCGGAAGCCTTCAAAGCCTCAAATAAGAGACATACAAACCTCTTAGTTTC

tRNA-Tyr
(Am)Desmognathus fuscus           -GGTAAAGTGGCCGAA--------C-AGGTATTAAGTTGTAGCCTTAATAATAGAGGTTTAAATCCTCTCTGTACC
(Am)Pachyhynobius shangchengens   -GGTAGAATGGCCGAA-------TT-AGGCGTAGGGTTGTAGCCCCTTTCATAAAGGTTAAAGTCCTTTTTTTACC
(Am)Pelobates cultripes           -GGTAAAATGGCCGAGAG-----CT-AGGCGGCAGGTTGTAGCCCTGATTACAAAGGTTTAAGTCCTTTTTTTACC
(Bi)Anser albifrons               -GGTAAGATGGCTGAGTG----TTG-AAGCGTTAGGCTGTAGTCCTTTTTACAGAGGTTTAATTCCTCTTCTTATC
(Bi)Ardea novaehollandiae         -GGTAAGATGGCTGAGTG----TTATAGGCGTTAGGCTGTAGTCCTTTTTACAGAGGTTTAATTCCTCTTCTTGCC
(Bi)Gallus varius                 -GGTAAGATGGCTGAGTG----TTG-AAGCGTTAGGCTGTAGTCCTTTTTACAGAGGTTCAATTCCTCTTCTTATC
(Ch)Centruroides limpidus         GGTTTGCGTGGCCGAAAT----CTCCAGGCATAAAACTGTAAATTTTATTATGAAACCTTT-CCCAAACCA-----
(Ch)Ixodes persulcatus            --GTAAAATGGCTGAAGA----AATTAAGCTATAAATTGTAAATTTATTGATGAAGA---AAATTCTTTTACT---
(Ch)Mesobuthus gibbosus           -GTTTGAGTAGCTTAAAC----ATC-AAGCATAAAACTGTAAATTTTACTATA-----TCT-AACAAATC------
(Cr)Gonodactylus chiragra         -GATAAAATGGCTGAG-------CTTAGGCGATAGATTGTAAATCTATTAACG-AGGTAAGAACTCCTCTTTTGTC
(Cr)Lepeophtheirus salmonis       -AATTAAGTAGTTTAA-------TAAAAACCTTAAATTGTAAATTTAAAATTAGATTTTC--TCTTAATTA-----
(Cr)Lysiosquillina maculata       -GATAAGGTGGCTGAGA------TTTAAGCGGTAGATTGTAAATCTATTAACG-AGG---GGTGTCCTCCTTTATC
(Fi)Coilia nasus                  -GGTAGGTTGGCCGAGAG----TTT-AGGCGGCGGATTGTAGCTCCGATTACAGAGGTTTAAGTCCTCTTTCTACC
(Fi)Salanx ariakensis             -GGTAAGATGGCCGAA-------GC-AAGCGGTGGATTGTAGCCCCATATATAGAGGTTTGAGTCCTCTTCTTACC
(Fi)Scleropages formosus          -GGTAGAATGGCTGAGTG----TTT-AAGCGGTGGGTTGTAGCCCCACACATGGAGGTTTAATTCCTCTTTCTATC
(He)Campodea lubbocki             -GATATGATGGCTGA-----A---ATAAGTGTTAGATTGTAAATCTATTTATGGTTA-ATAACCTCATATCA----
(He)Pteronarcys princeps          -GGTAGAATGGCTGAGA-----TATAAGGTGATAGATTGTAAATCTATTTA-GGAGGTATA--CCCCTCTTCTACC
(He)Reticulitermes virginicus     -AGTGGAGTGGCTGAGACAGATAAGTAGGCGGTAGGCTGTAAATCTATTTAGGAGGTTAGTACGTGACTCTTCCAC
(Ma)Elephas maximus               -GGTAAAATGGCTGAG--------T-AAGTATTAGACTGTAAATCTAACCACAGAGA-GTAA--TCTCTTTTTACC
(Ma)Eubalaena australis           -GGTAAAATGGCCGAG--------T-AGGCATTAGACTGTAAATCTAAAGACAGAGGTTGAAGTCCTCTTTTTACC
(Ma)Phascolarctos cinereus        -GGTAAAATGGCTGAGG-------T-AAGCATTGAATTGTAAATTCAAAGACAGAGGTATAA--TCTCTTTTTACC
(Mo)Loligo bleekeri               -TATGAGGTGGTCGAT-------TAAAGTCGGTAGATTGTAAATCTATGAATAAGTTGTAGGACTTTCTCATG---
(Mo)Mytilus galloprovincialis     -CATAGGATGGCCGAGGA-----AT-AGGTGGTGAGCTGTAAACTCATAAACA-AGGCGTAAGCCTTTCTTATGA-
(Mo)Roboastra europaea            -ATTTTGATGGCTGA-----ATTAATAAGCGATAGATTGTAAATCTACTAATGGTTGTATAACCTCACTGTA----
(Re)Indotestudo elongata          -GGTAAGATGGCTGAATG----TCC-AAGCGTTAGGCTGTAGACCTTTTTATAGGGGTTTAATTCCTCTTCTTATC
(Re)Pantherophis slowinskii       -GGTAGTATGGCTGATTA----AA----GCGGTAATTTGTAAAATTATGTATAGGAT---GTTCCTTGCTATCA--
(Re)Pelomedusa subrufa            -AGTGAGATGACCGAGTG----TTC-AGGTGTTAGGTTGTAAACCTTTTTATAGAGGTTTAATCCCTCTTCTTACT
```

Fig. (S1). Alignment of anticodons of 22 tRNA genes from animal mtDNAs. Among animal mitochondrial genomes, the anticodons of tRNA genes are highly conserved (framed in green). We chose taxonomically diverse animals as representatives of different lineages.

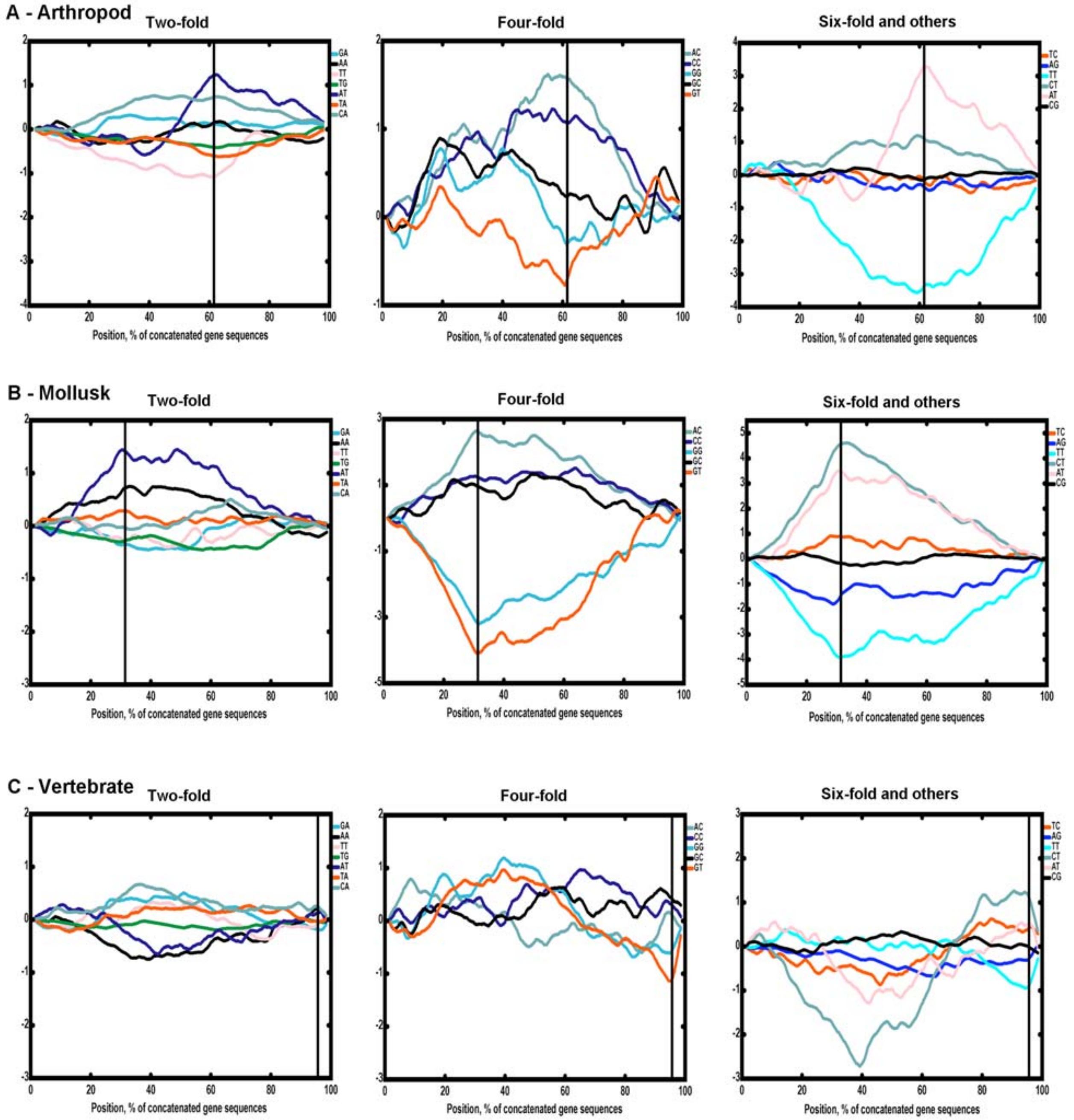

Fig. (S2). The dinucleotide frequency of cp1 and cp2 of concatenated 13 protein-coding sequences of mitochondrial genomes from diverse animal taxa: **A**, *Pyrocoelia rufa*, a representative of arthropods; **B**, *Lampsilis ornate*, a representative of mollusks; and **C**, *Trichosurus vulpecula*, a representative of vertebrates. Numbers on the y-axis indicate the relative abundance of dinucleotide sequences. The vertical line indicates the boundary of the L- and H-strand genes.
